# High Temperature, Living Polymerization of Ethylene by a Sterically-Demanding Nickel(II) α-Diimine Catalyst

**DOI:** 10.3390/polym10010041

**Published:** 2018-01-02

**Authors:** Lauren A. Brown, W. Curtis Anderson, Nolan E. Mitchell, Kevin R. Gmernicki, Brian K. Long

**Affiliations:** Department of Chemistry, University of Tennessee, Knoxville, TN 37996, USA; labrown2436@gmail.com (L.A.B.); wander24@vols.utk.edu (W.C.A.J.); nmitch0823@gmail.com (N.E.M.); krgmer6910@gmail.com (K.R.G.)

**Keywords:** polyethylene, living polymerization, nickel α-diimine, catalysis

## Abstract

Catalysts that employ late transition-metals, namely Ni and Pd, have been extensively studied for olefin polymerizations, co-polymerizations, and for the synthesis of advanced polymeric structures, such as block co-polymers. Unfortunately, many of these catalysts often exhibit poor thermal stability and/or non-living polymerization behavior that limits their ability to access tailored polymer structures. Due to this, the development of catalysts that display controlled/living behavior at elevated temperatures is vital. In this manuscript, we describe a Ni α-diimine complex that is capable of polymerizing ethylene in a living manner at temperatures as high as 75 °C, which is one of the highest temperatures reported for the living polymerization of ethylene by a late transition metal-based catalyst. Furthermore, we will demonstrate that this catalyst’s living behavior is not dependent on the presence of monomer, and that it can be exploited to access polyethylene-based block co-polymers.

## 1. Introduction

Controlled/living polymerizations offer a precise means by which polymer structure, co-monomer incorporation levels, and even regio- and stereoselectivity can be tailored [1,2,3,4,5,6,7]. Living polymerization methodologies are those that are free of deleterious chain termination and irreversible chain transfer events [8]. Specifically for ethylene and α-olefin polymerizations, these chain transfer and termination events are often suppressed at low temperatures, and it is for this reason that most living polymerizations of ethylene occur at or below ambient temperature [1,9,10,11,12,13,14]. However, due to the growing demand for polymers with tailored structure and because industrial olefin polymerizations are often conducted at elevated temperatures (70–115 °C), the overall utility of most reported late transition metal-based olefin polymerization catalysts are severely limited [15,16].

**Figure 1:**
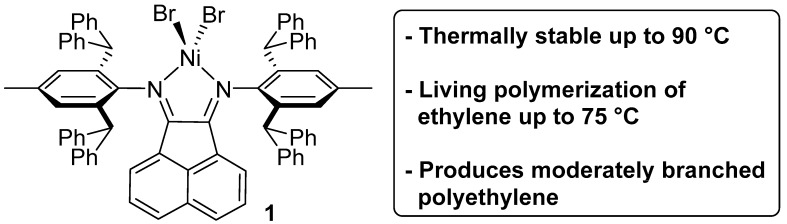
Catalyst **1** used for ethylene polymerizations.

To date, only a few examples of Ni- and Pd-based olefin polymerization catalysts that exhibit controlled/living polymerization behavior at superambient temperatures have been reported [17,18,19,20,21,22]. Furthermore, most of these polymerizations are not performed using ethylene as a sole feedstock, but rather utilize higher α-olefins. This lack of Ni- and Pd-based catalysts capable of performing living ethylene polymerizations at elevated temperatures represents a fundamental gap in current knowledge. To address this issue, we report herein that the Ni-based α-diimine catalyst **1** readily polymerizes ethylene in a living fashion at temperatures as high as 75 °C (Figure 1). To the best of our knowledge, this is one of the highest temperature living ethylene polymerizations using a Ni- or Pd-based catalyst reported to date.

## 2. Materials and Methods

All experiments were performed under a dry nitrogen atmosphere using standard Schlenk techniques or an MBraun inert-atmosphere glove box (Stratham, NH, USA), unless otherwise noted. Solvents were purified using a two-column solid-state Innovative Technologies PureSolv Solvent Purification System (Amesbury, MA USA) and degassed via three freeze-pump-thaw cycles prior to use. NMR solvents were purchased from Cambridge Isotope Laboratories (Andover, MA, USA). Catalyst **1** was prepared according to literature [23]. Liquid chromatography-mass spectrometry (LC-MS) experiments were performed using a Thermo Fisher Scientific Exactive (Waltham, MA, USA) Plus Orbitrap MS (Waltham, MA, USA) using direct injection, full-scan, electrospray ionization. All polymerizations were activated using methylaluminoxanes (PMAO-IP) that was purchased from the Akzo Nobel (Amsterdam, the Netherlands) and used as received. All other reagents were purchased from commercial vendors and used without further purification. Gel permeation chromatography (GPC) was performed at 160 °C in 1,2,4-trichlorobenzene at a flow rate of 1.0 mL/min using a Malvern Viscotek HT-GPC (Malvern, UK) equipped with triple detection. Polymer ^1^H NMR spectra were obtained in CDCl_3_ using a Bruker 400 MHz NMR at 50 °C. All NMR spectra are referenced relative to their residual solvent signal. Branching content was determined by ^1^H NMR spectroscopy using the formula (CH_3_/3)/((CH + CH_2_ + CH_3_)/2) × 1000 [24]. Polymer thermal transition temperatures (*T*_m_) were measured using a TA instruments Q2000 Differential Scanning Calorimeter (DSC, New Castle, DE, USA) and recorded on the second heating cycle at a heating rate of 10 °C/min. Polyethylene samples for tensile testing were melt-pressed using 0.5 g of polyethylene sample in a Carver Press at 10,000 lbs. of pressure for 10 minutes, followed by slow cooling to room temperature. Both faces of the Carver Press were covered with Kapton film prior to pressing to prevent film sticking and contamination. Tensile (stress vs. strain) analysis was performed using an Instron 5943 electro mechanic single column universal testing machine (Norwood, MA, USA) with pneumatic tension grips connected to a 100 N load cell at a strain rate in accordance with the ASTM D638 standard.

General ethylene polymerization procedure: Under an inert atmosphere, a Fisher-Porter bottle was charged with catalyst **1** (5 µmol) dissolved in dichloromethane (DCM) (2 mL), toluene (98 mL), and a magnetic stir bar. The Fisher-Porter bottle was sealed and placed in an oil bath set to the desired temperature. The vessel was pressurized with ethylene gas while stirring and equilibrated for 10 min. PMAO-IP (100 equiv) was injected to initiate the polymerization and the polymerization was stirred continuously for the desired time. All polymerizations were quenched via the addition of methanol (MeOH) (10 mL). The polymer was precipitated using excess acidic MeOH (5% HCl in MeOH) and allowed to stir in that solution for 24 h. The resultant polymer was filtered and dried to constant weight in a vacuum oven.

## 3. Results and Discussion

### 3.1. Synthesis of High Purity Catalyst ***1***

Catalyst **1** was previously reported by our group and found to exhibit significantly enhanced time-resolved thermal stability due to its acenaphthenequinone-based ligand that contains sterically-demanding *N*-aryl moieties [23,25]. More specifically, catalyst **1** displayed virtually perfect thermal stability up to reaction temperatures as high as 90 °C, which is one of the highest temperatures reported for α-diimine ligated Ni-based catalyst. As a note, these bulky *N*-aryl moieties have been successfully utilized by other researchers to produce a variety catalysts with unique and impressive behavior [26,27,28,29]. However, despite the remarkable thermal stability of catalyst **1**, we noted that the polymer produced often exhibited a small, high molecular weight shoulder when analyzed by gel permeation chromatography (GPC) (see Supplementary Materials, Figures S1 and S28). This high molecular weight shoulder resulted in polymer samples with molecular weight dispersities broader than typically encountered for a controlled/living polymerization, albeit still relatively narrow as compared to many commonly employed olefin polymerization catalysts [25].

After further investigation, we have now been able to attribute this high molecular weight shoulder to a catalyst impurity that was not detectable via initial ^1^H NMR spectroscopy. More specifically, we discovered that the problematic impurity was an asymmetric analogue of catalyst **1** in which only three benzhydryl (*i*Pr*) *N*-aryl moieties are present. This asymmetric ligand precursor was identified via liquid chromatography-mass spectrometry (LC-MS) prior to metalation and is a result of incomplete conversion during the synthesis of the bulky 2,6-disubstituted aniline moiety (see Supplementary Materials, Figures S1 and S2).

Ultimately, high-purity Ni complex **1** was synthesized according to our previously reported procedure [25]. However, in order to eliminate any undesired monosubstituted ligand impurities, which result during the Friedel-Crafts alkylation of *p*-toluidene using benzhydrol (diphenylmethanol) and ZnCl_2_ [29], the reaction product was recrystallized in quadruplicate from isopropanol. These additional recrystallizations completely removed any traces of monosubstituted aniline, which was confirmed via LC-MS (see Supplementary Materials, Figure S3). This high-purity aniline was then condensed onto acenaphthenequinone using a two-step, one-pot reaction and subsequently metalated using NiBr_2_ (dimethoxyethane adduct) to yield high-purity catalyst **1**, which is used exclusively in the following studies. 

### 3.2. Ethlene Polymerizations using High-Purity ***1***/PMAO-IP at Elevated Temperatures

Ethylene polymerization trials were performed using catalyst **1** (5 µmol) and activated with PMAO-IP (100 equiv) (Table 1). Polymerization trials were conducted at 70, 75, and 80 °C and were monitored as a function of time up to 1 h. Catalyst **1** was found to be highly active over the entire 1 h trial (TOF = 6300–8700) even at low ethylene pressure (15 psi) and elevated temperatures (70–80 °C). The resultant polymers were analyzed via GPC and ^1^H NMR. The number average molecular weights (*M*_n_) of the polymers produced by **1**/MAO were found to increase steadily as a function of time reaching final molecular weights ranging from 193–253 kg/mol, depending on reaction temperature (Table 1). Polymer molecular weight dispersities (*Đ*) remained below 1.2 for polymerizations at both 70 and 75 °C, which suggested that this catalyst might indeed be living at these temperatures. In contrast, polymers produced by **1**/MAO at 80 °C exhibited broadened dispersities as a function of time (Table 1, entries 11–14) suggesting that **1**/MAO may deviate from living behavior at temperatures exceeding 75 °C. While our previous work established that catalyst **1** remains active at temperatures as high as 90 °C [25], the broadening dispersities observed at 80 °C are attributed to increased chain transfer or chain termination events. Lastly, ^1^H NMR analysis showed that all polymers produced by complex **1** contained 45–50 branches per 1000 total carbons (*B*) (see Supplementary Materials, Figures S4–S6).

**Figure 2:**
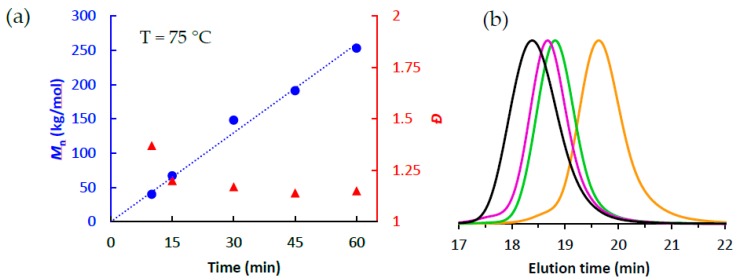
(**a**) Plot of *M*_n_ (●) and *Đ* (▲) as a function of polymerization time when using **1**/PMAO-IP at 75 °C; (**b**) GPC traces of polymerizations at 75 °C as a function of polymerization time (black = 60 min, purple = 45 min, green = 30 min, orange = 15 min).

To determine if catalyst **1** is, indeed, living at these elevated temperatures, we plotted their progression of molecular weight (*M*_n_) and dispersity (*Đ*) as a function of polymerization time. Figure 2 clearly shows that catalyst **1** provides a linear increase in polyethylene molecular weight at 75 °C, ultimately reaching over 250 kg/mol after 1 h. Moreover, GPC analysis also demonstrated that the polymers produced at 75 °C remained monomodal during the course of polymerization and their dispersities narrowed as the polymerization progressed (*Đ* = 1.37→1.15) (Table 1, entries 6–10). Both the observed linear increase in molecular weight and decreasing dispersity as a function of time strongly support that catalyst **1** is indeed living for polymerizations at 75 °C. The same trends can be seen for polymerizations at 70 °C (see Supplementary Materials, Figures S7 and S8).

**Figure 3:**
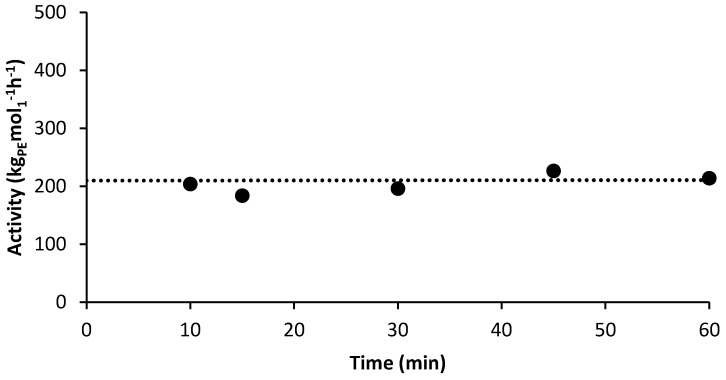
Plot of activity versus reaction time for ethylene polymerizations using **1**/PMAO-IP at 75 °C.

To further emphasize the controlled/living character of catalyst **1**, its polymerization activity at 75 °C was calculated and plotted as a function of time to ensure that no loss in catalytic activity was observed. As shown in Figure 3, catalyst **1** retains constant activity during ethylene polymerizations at 75 °C for the full time monitored (see Supplementary Materials, Figure S9). From these results and from those presented in Figure 2, we conclude that catalyst **1** does, in fact, polymerize ethylene in a controlled/living manner at both 70 and 75 °C. To the best of our knowledge, this is one of the highest temperatures reported for the living polymerization of ethylene mediated by any late transition metal-based catalyst.

**Figure 4:**
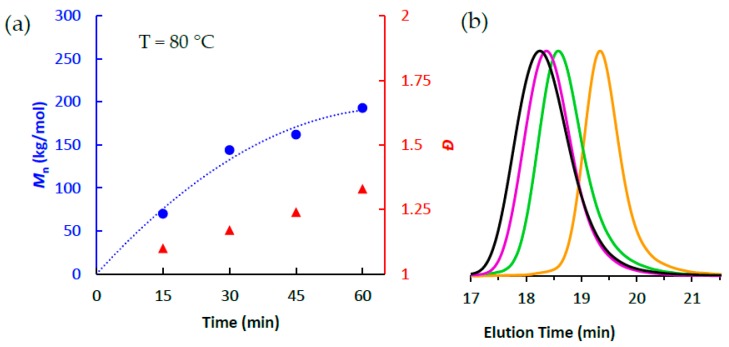
(**a**) Plot of *M*_n_ (●) and *Đ* (▲) as a function of polymerization time using **1**/PMAO-IP at 80 °C; and (**b**) GPC traces of polymerizations at 75 °C as a function of polymerization time (black = 60 min, purple = 45 min, green = 30 min, orange = 15 min).

In contrast to the results observed at 75 °C, ethylene polymerizations using **1**/MAO at 80 °C (Table 1, entries 11–12) were found to deviate from linear molecular weight growth after just 30 min of polymerization, and polymer dispersity values steadily increased over the 1 h polymerization (*Đ* = 1.10→1.33) (Figure 4a). This was also evidenced by their respective GPC traces in which the resultant polymer samples broadened as a function of polymerization time (Figure 4b). Therefore, we conclude that while **1**/MAO maintains living polymerization behavior at 75 °C, it begins to diverge at temperatures ≥ 80 °C (see Supplementary Materials, Figure S10). Lastly, we would like to note that though catalyst **1** does not meet the criteria of “living” at these higher temperatures (≥80 °C), our previous studies have conclusively shown that it does remain active for ethylene polymerizations up to 90 °C [25]. This suggests that while catalyst **1** does not fit the most rigorous definitions of “living” at these temperatures, catalyst decomposition/deactivation is minimal and deviations from ideal living behavior for polymerizations conducted between 80–90 °C are likely prone to undesirable chain transfer and/or termination rather than catalyst deactivation.

### 3.3. Evaluating the Livingness of Catalyst ***1***/PMAO-IP in the Absence of Monomer

**Figure 5:**
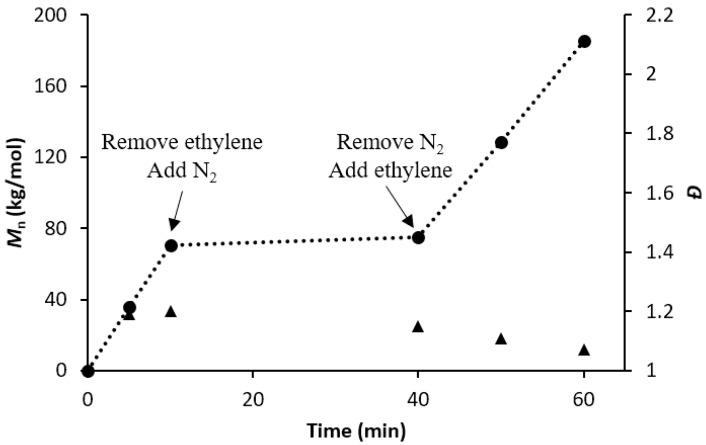
Plot of *M*_n_ as a function of polymerization time using **1**/PMAO-IP at 75 °C.

To examine the utility of living catalyst **1**/PMAO-IP at elevated temperatures, and to determine if living behavior is retained in the absence of monomer, we conducted an “on-off-on” switching experiment at 75 °C (Figure 5). Therein, catalyst **1** was initiated at 75 °C using PMAO-IP and allowed to polymerize for 10 min under 15 psi of ethylene feed pressure. After the allotted time, ethylene was purged from the reaction vessel and replaced by an inert nitrogen atmosphere, which was maintained for 30 min. After those 30 min, the system was re-charged with ethylene (15 psi) to continue the polymerization. During the polymerization periods, aliquots were removed at 5, 10, 40, 50, and 60 min to monitor the polymerization behavior by **1**/PMAO-IP.

The results presented in Figure 5 show that polyethylene molecular weight increased steadily when monomer was present, but that any chain-extension halted after ethylene was evacuated from the reactor. Reintroduction of ethylene to the reaction vessel resulted in polyethylene chain-extension, ultimately reaching molecular weights that exceeded 150 kg/mol. The molecular weight of the polyethylene increased linearly when ethylene monomer was present, and its molecular weight dispersity remained low throughout the entire polymerization. This “on-off-on” experiment (a) further supports **1**/PMAO-IP’s ability of polymerizing ethylene in a living fashion at elevated temperatures; and (b) provides strong evidence that the livingness of **1**/PMAO-IP is not dependent on the presence of excess ethylene monomer.

### 3.4. Synthesis of Block Copolyethylenes by ***1***/PMAO-IP

Due to catalyst **1**’s ability to: (a) polymerize ethylene in a living fashion over a wide range of temperatures (−40–75 °C) [23]; and (b) produce polyethylenes with low branching density at low temperatures and polyethylenes with higher branching density at higher temperatures, we hypothesized that block co-polymers could be synthesized. These block co-polymers would be synthesized via modulation of reaction temperature, which would result in block copolymer segments that vary in PE branching content. The ability to control polyethylene branching content via reaction temperature is well known when using Ni α-diimine catalysts, and is caused by the catalyst propensity to chain-walk to a greater or lesser extent as a function of temperature [30,31].

Synthesis of polyethylene-based diblock and triblock co-polymers was performed using **1**/PMAO-IP and modulating the temperature of the reactor between −40 and 75 °C. As can be seen in Table 2 (entry 1) ethylene polymerizations conducted at −40 °C produce highly linear PE with a *T*_m_ = 128.5 °C. In contrast, polymerizations conducted at 75 °C produce PE with greater branching density as evidenced by its lower melting temperature (*T*_m_ = 62.4 °C) (Table 2, entry 2). To access diblock co-polymers, polymerizations were initiated at −40 °C and the reactor maintained at this temperature for 25 min before being transferred to an oil bath thermostated at 75 °C, where the polymerization continued for an additional 45 min (Table 2, entry 3). Triblock co-polymers were accessed in an analogous manner to diblocks, except that a third block segment was produced by transferring the polymerization reactor to a cold bath at −40 °C for an additional 25 min (Table 2, entry 4) resulting in a linear-branched-linear block copolymer structure. As can be seen in Table 2, the molecular weights of the synthesized diblock and triblock co-polyethylenes increased as expected from with the addition of polymer blocks, and low molecular weight dispersity values were obtained in all cases (Table 2). Furthermore, the block co-polymers produced using **1**/PMAO-IP displayed the two melting transition values (*T*_m_), which indicates that both branched polyethylene segments and highly-linear polyethylene segments are present.

Since both the high-temperature and low-temperature segments of these diblock and triblock co-polymers are each crystalline, we hypothesized that no thermoplastic elastomeric behavior would be observed. To test this hypothesis, the mechanical properties of these block co-polymers were evaluated. Each polymer from Table 2 was melt-pressed into samples for tensile (stress-strain) testing. Those tests showed that the mostly linear homopolymer produced by catalyst **1**/PMAO-IP at −40 °C (Table 2, entry 1) had a Young’s modulus (*E*) of 120.5 MPa, which indicates a more rigid polymer that is expected from a polyethylene sample that contains very little branching. In contrast, the more branched homopolymer produced at 75 °C displayed a Young’s modulus (*E*) of 13.5 MPa (Table 2, entry 2), which indicates a much less rigid material and a polyethylene sample that contains a higher branching content. Both the diblock and the triblock co-polymers contain segments of each of these branching contents and have a Young’s modulus of approximately 40 MPa, which is expected for co-polymers containing both moderately-branched and low-branched segments that are both crystalline.

## 4. Conclusions

The sterically-demanding Ni α-diimine catalyst **1** has been shown to polymerize ethylene in a living fashion at reaction temperatures significantly above room temperature. More specifically, complex **1** exhibits living behavior up to 75 °C when activated with PMAO-IP, which is evidenced by linearly increasing molecular weights and narrowing polymer dispersities as a function of polymerization time and conversion. This living behavior was further emphasized via “on-off-on” polymerization experiments that provide strong evidence that the living nature of this catalyst is not dependent on the presence of excess monomer, and that chain extension can be resumed upon further monomer addition (Figure 5). To the best of our knowledge, this is one of the highest temperatures reported for the living polymerization of ethylene using a late transition-metal complex to date.

We have exploited the living behavior of **1**/PMAO-IP at elevated temperatures to produce polyethylene-based block co-polymers in which the block copolymer segments differ in regards to their branching content. This was accomplished by manipulating the propensity of **1**/PMAO-IP to undergo chain-walking at different temperatures and the living nature of this catalyst at both high and low temperatures. Lastly, we have provided strong evidence that though catalyst **1**/PMAO-IP deviates from living polymerization behavior at temperatures ≥80 °C, the catalytically-active species remains active and thermally stable to temperatures as high as 90 °C. We attribute this deviation from living behavior to undesirable chain transfer and chain termination events.

## Figures and Tables

**Table 1:** Ethylene polymerizations using catalyst **1** ^a^.

Entry	Time (min)	*T*_rxn_ (°C)	Yield (g)	TOF ^b^	*M*_n_^c^ (kg/mol)	*Đ* ^c^	*B* ^d^
1	10	70	0.16	6900	44	1.35	47
2	15	70	0.22	6300	50	1.22	48
3	30	70	0.49	7000	128	1.14	48
4	45	70	0.77	7300	164	1.18	47
5	60	70	0.96	6900	244	1.17	48
6	10	75	0.17	7300	40	1.37	47
7	15	75	0.23	6600	67	1.20	49
8	30	75	0.49	7000	155	1.17	47
9	45	75	0.85	8100	191	1.14	50
10	60	75	1.07	7600	253	1.15	46
11	15	80	0.30	8600	70	1.10	48
12	30	80	0.61	8700	114	1.17	49
13	45	80	0.72	6900	162	1.24	50
14	60	80	0.93	6600	193	1.33	45

^a^ Ethylene polymerization conditions: 5.0 μmol of catalyst **1**, 15 psi of ethylene, 98 mL of toluene, 2 mL of dichloromethane, and 100 equiv of PMAO-IP; ^b^ Turnover frequency (TOF) = mol of ethylene/(mol of cat. × h); ^c^ Determined using triple detection gel permeation chromatography at 160 °C in 1,2,4-trichlorobenzene (see Supplementary Materials, Figures S11–S24). ^d^ Branches per 1000 total carbons determined via ^1^H NMR.

**Table 2:** Synthesis of PE and temperature modulated block co-polyethylene by **1**/PMAO-IP ^a^.

Entry	*t*_1_ (min)/T_1_ (°C)	*t*_2_ (min)/*T*_2_ (°C)	*t*_3_ (min)/*T*_3_ (°C)	*M*_n_^b^*,* Tot (kg/mol)	*Đ* ^b^	*T*_m_^c^ (°C)	*E* ^d^ (MPa)
1	25/−40	-	-	52	1.22	128.5	120.5 ± 3.8
2	45/75	-	-	191	1.14	62.4	13.5 ± 5.9
3	25/−40	45/75	-	279	1.26	61.3, 113.5	40.4 ± 3.0
4	25/−40	45/75	25/−40	352	1.23	66.2, 111.0	40.7 ± 8.2

^a^ Ethylene polymerization conditions: 5.0 μmol of catalyst **1**, 15 psi of ethylene, 98 mL of toluene, 2 mL of dichloromethane, and 100 equiv. of PMAO-IP; ^b^ Determined using triple detection gel permeation chromatography at 160 °C in 1,2,4-trichlorobenzene(see Supplementary Materials, Figures S25–S27); ^c^ Determined by differential scanning calorimetry, second heating cycle (see Supplementary Materials, Figures S29–S32); ^d^ Determined from stress-strain data of PE films (see Supplementary Materials, Figure S33).

## Supplementary Materials

The following are available online at www.mdpi.com/2073-4360/10/1/41/s1, Figure S1: Modified synthetic route to obtain complex 1 in ultrahigh purity; Figure S2: LC-MS of bulky *i*Pr* aniline in DCM prior to purification; Figure S3: LC-MS of bulky *i*Pr* aniline in DCM after four recrystallizations from isopropanol; Figure S4: ^1^H NMR spectrum of polyethylene at 70 °C. (Table 1, entry 3); Figure S5: ^1^H NMR spectrum of polyethylene at 75 °C. (Table 1, entry 8); Figure S6: ^1^H NMR spectrum of polyethylene at 80 °C. (Table 1, entry 11); Figure S7: (a) Plot of *M*_n_ and *Đ* as a function of polymerization time using 1/PMAO-IP at 70 °C; (b) GPC traces of polymerizations run at 70 °C at various polymerization times; Figure S8: Plot of *M*_n_ and *Đ* as a function of polymer yield using 1/PMAO-IP at 70 °C; Figure S9: Plot of *M*_n_ and *Đ* as a function of polymer yield using 1/PMAO-IP at 75 °C; Figure S10: Plot of *M*_n_ and *Đ* as a function of polymer yield using 1/PMAO-IP at 80 °C; Figure S11: GPC of polyethylene. (Table 1, entry 1); Figure S12: GPC of polyethylene. (Table 1, entry 2); Figure S13: GPC of polyethylene. (Table 1, entry 3); Figure S14: GPC of polyethylene. (Table 1, entry 4); Figure S15: GPC of polyethylene. (Table 1, entry 5); Figure S16: GPC of polyethylene. (Table 1, entry 6); Figure S17: GPC of polyethylene. (Table 1, entry 7); Figure S18: GPC of polyethylene. (Table 1, entry 8); Figure S19: GPC of polyethylene. (Table 1, entry 9); Figure S20: GPC of polyethylene. (Table 1, entry 10); Figure S21: GPC of polyethylene. (Table 1, entry 11); Figure S22: GPC of polyethylene. (Table 1, entry 12); Figure S23: GPC of polyethylene. (Table 1, entry 13); Figure S24: GPC of polyethylene. (Table 1, entry 14); Figure S25: GPC of polyethylene. (Table 2, entry 1); Figure S26: GPC of polyethylene. (Table 2, entry 3); Figure S27: GPC of polyethylene. (Table 2, entry 4); Figure S28: Representative GPC of polyethylene produced using complex 1 prior to rigorous ligand purification; Figure S29: DSC of polyethylene. (Table 2, entry 1); Figure S30: DSC of polyethylene. (Table 2, entry 2); Figure S31: DSC of polyethylene. (Table 2, entry 3); Figure S32: DSC of polyethylene. (Table 2, entry 4); Figure S33: Plot of Stress versus Strain for polyethylene homopolymers and block co-polymers.
