# Supplementary material for: High Temperature, Living Polymerization of Ethylene by a Sterically-Demanding Nickel(II) α-Diimine Catalyst
Source: Polymers (Basel). 2018 Jan 2;10(1):41. doi: 10.3390/polym10010041 (PMC6415045; doi:10.3390/polym10010041)
Supplement: Supplementary file 1 [file polymers-10-00041-s001.pdf]

Supporting Information for:

High Temperature, Living Polymerization of Ethylene by a Sterically Demanding Nickel(II) α-Diimine Catalyst

Lauren A. Brown, W. Curtis Anderson Jr., Nolan E. Mitchell, Kevin R. Gmernicki, and Brian K. Long*

Department of Chemistry, University of Tennessee, Knoxville, TN 37996

e-mail: long @utk.edu

Table of Contents

Modified synthetic scheme S2
Liquid chromatography-mass spectra S2
NMR spectroscopy S3
Graphs of polymer M_n and $Đ$ using **1**/PMAO-IP S5
GPC data for obtained PE S7
DSC data for obtained PE S25
Stress versus Strain data for PE S27

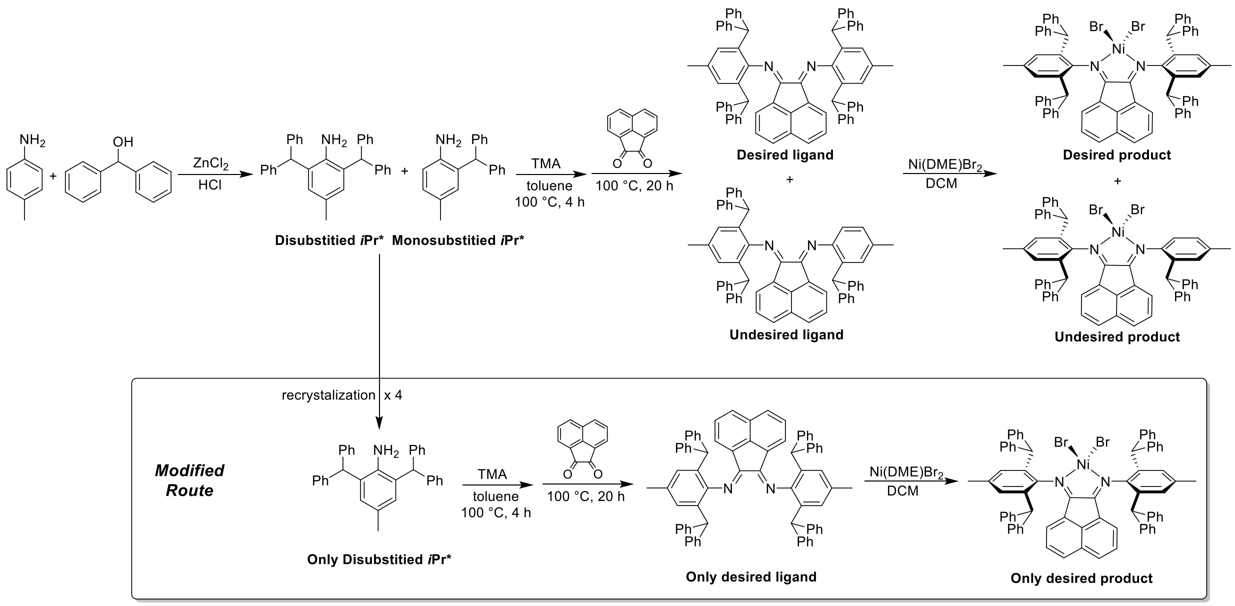

Figure S1. Modified synthetic route to obtain complex **1** in ultrahigh purity.

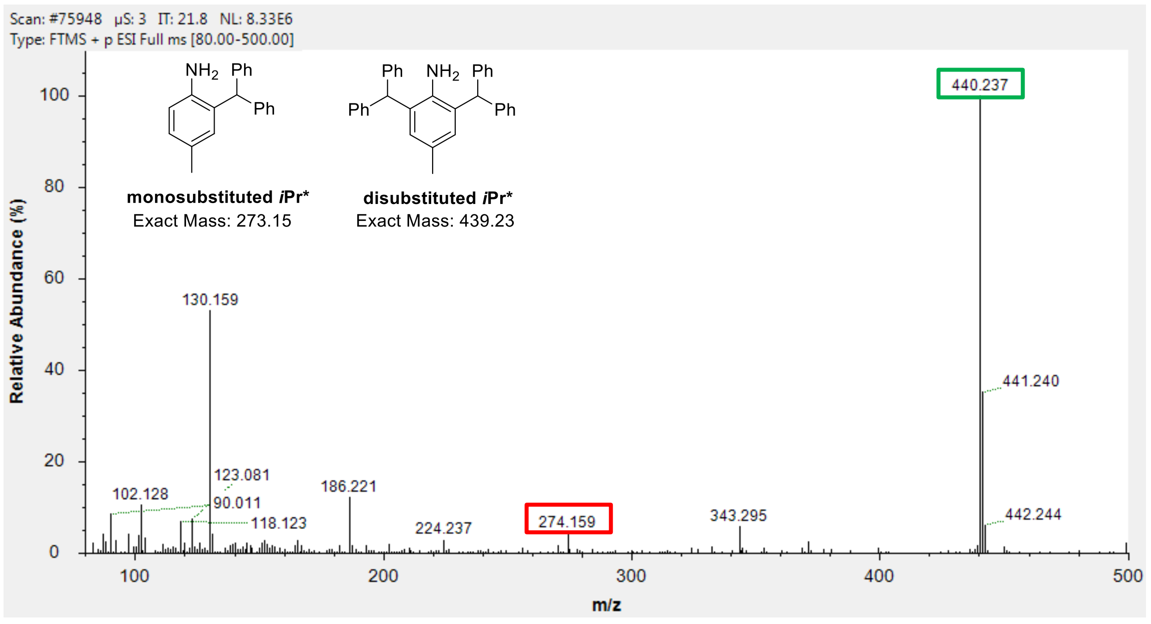

Figure S2. LC-MS of bulky *i*Pr* aniline in DCM prior to purification.

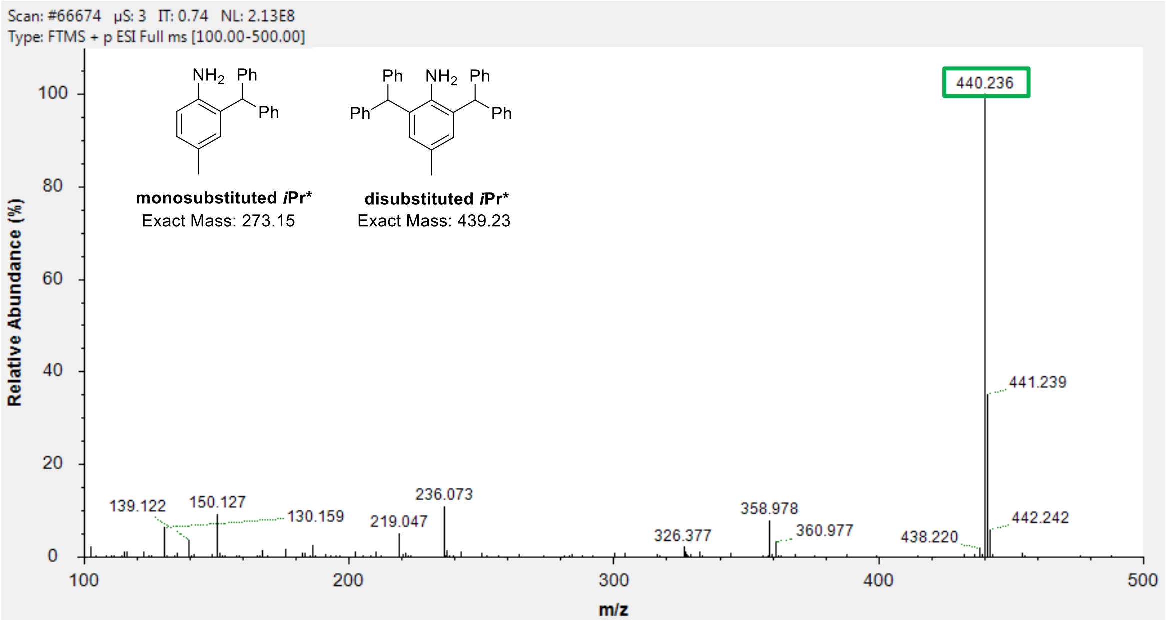

Figure S3. LC-MS of bulky *i*Pr* aniline in DCM after four recrystallizations from isopropanol.

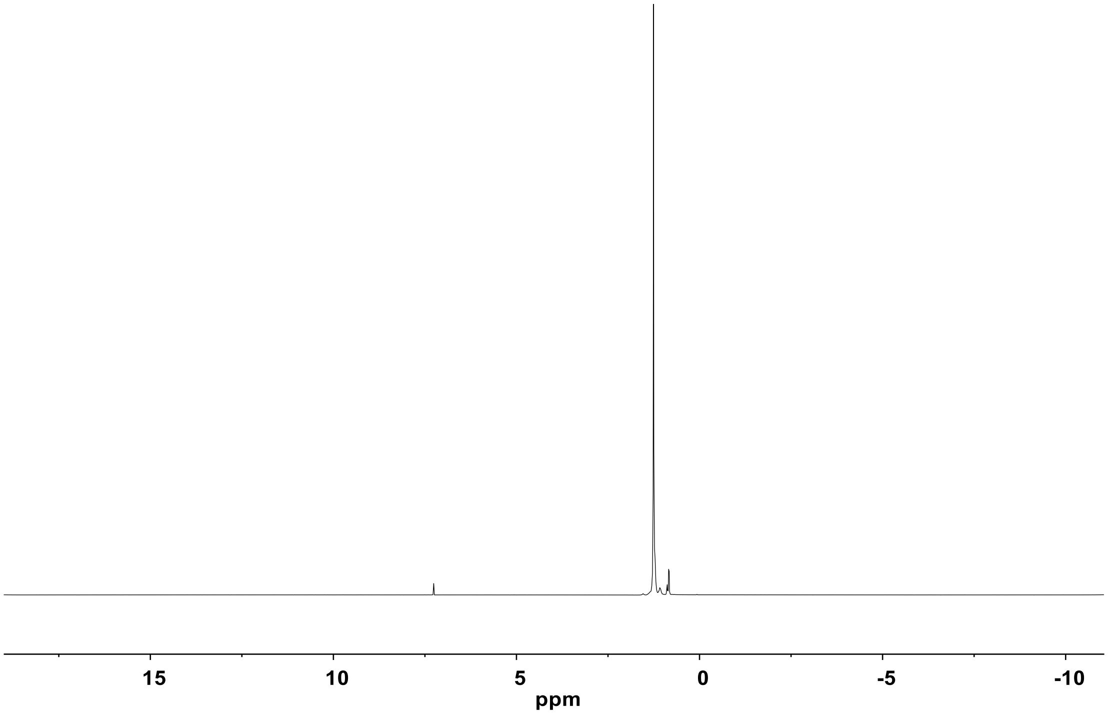

Figure S4. ^{1}H NMR spectrum of polyethylene at 70 °C. (Table 1, Entry 3).

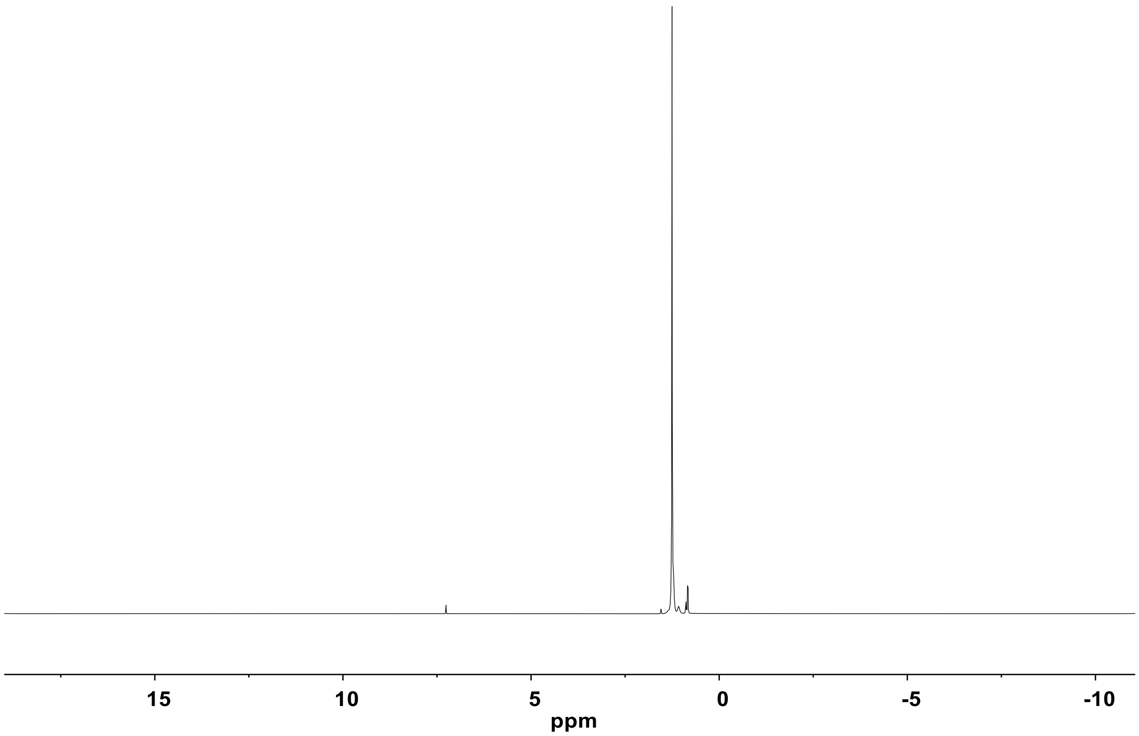

Figure S5. ^{1}H NMR spectrum of polyethylene at 75 °C. (Table 1, Entry 8).

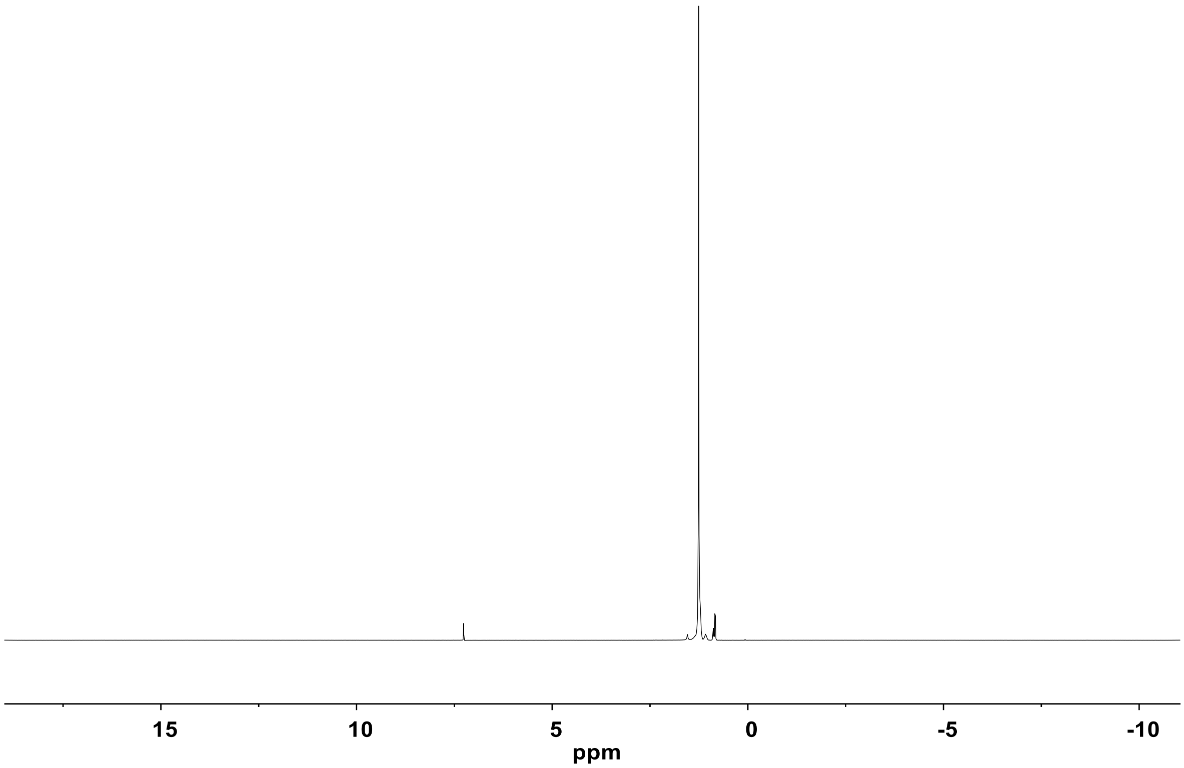

Figure S6. ^{1}H NMR spectrum of polyethylene at 80 °C. (Table 1, Entry 11).

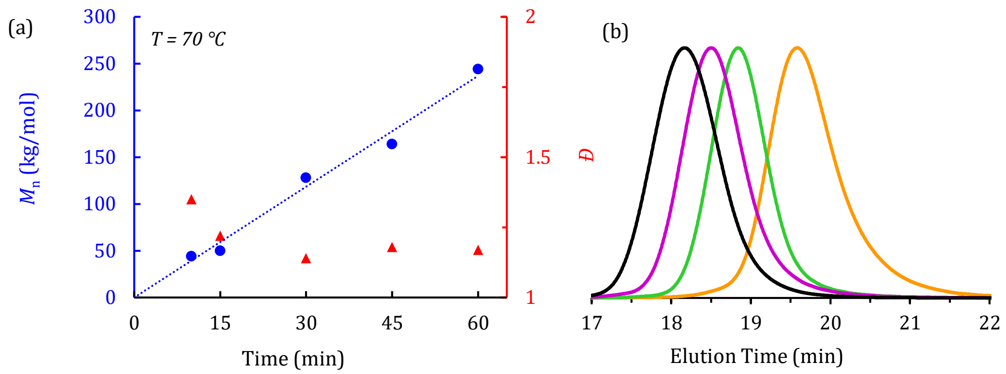

Figure S7. (a) Plot of M_n (blue circles) and *Đ* (red triangles) as a function of polymerization time using **1**/PMAO-IP at 70 °C. (b) GPC traces (viscometer detector) of polymerizations run at 70 °C at various polymerization times (black = 60 min, purple = 45 min, green = 30 min, orange = 15 min).

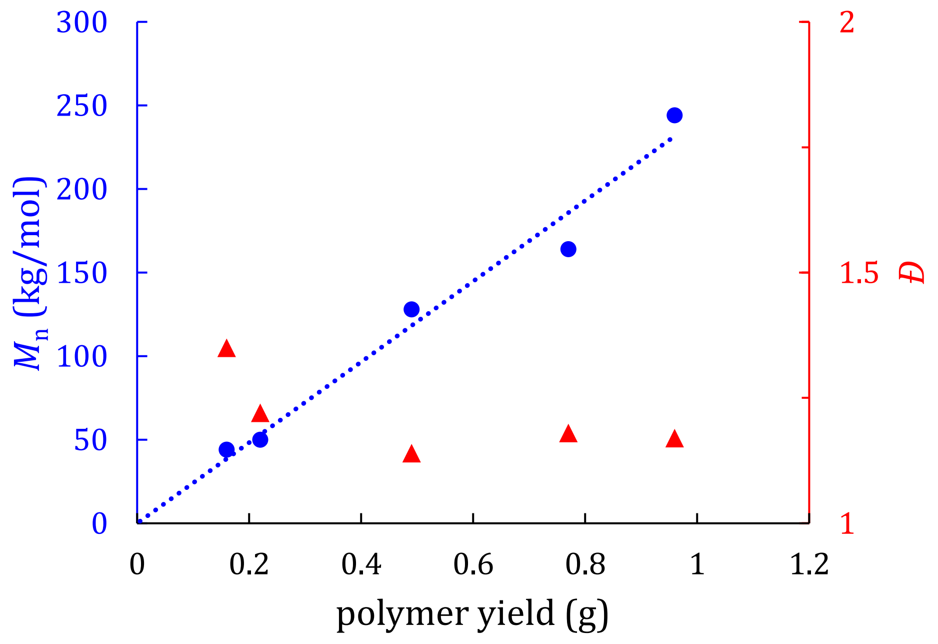

Figure S8. Plot of M_n (blue circles) and *Đ* (red triangles) as a function of polymer yield using **1**/PMAO-IP at 70 °C.

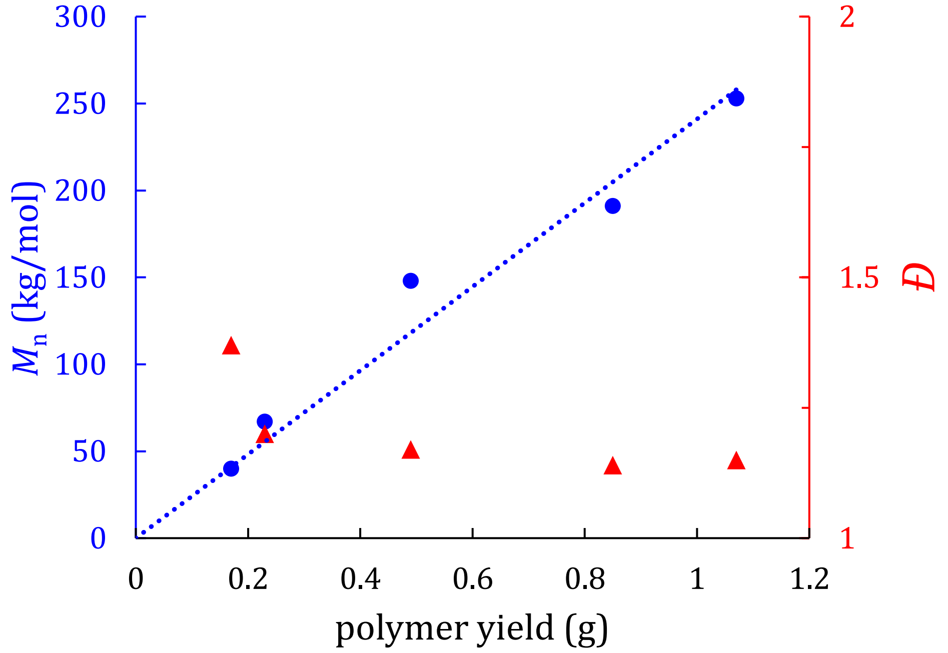

Figure S9. Plot of M_n (blue circles) and *Đ* (red triangles) as a function of polymer yield using **1**/PMAO-IP at 75 °C.

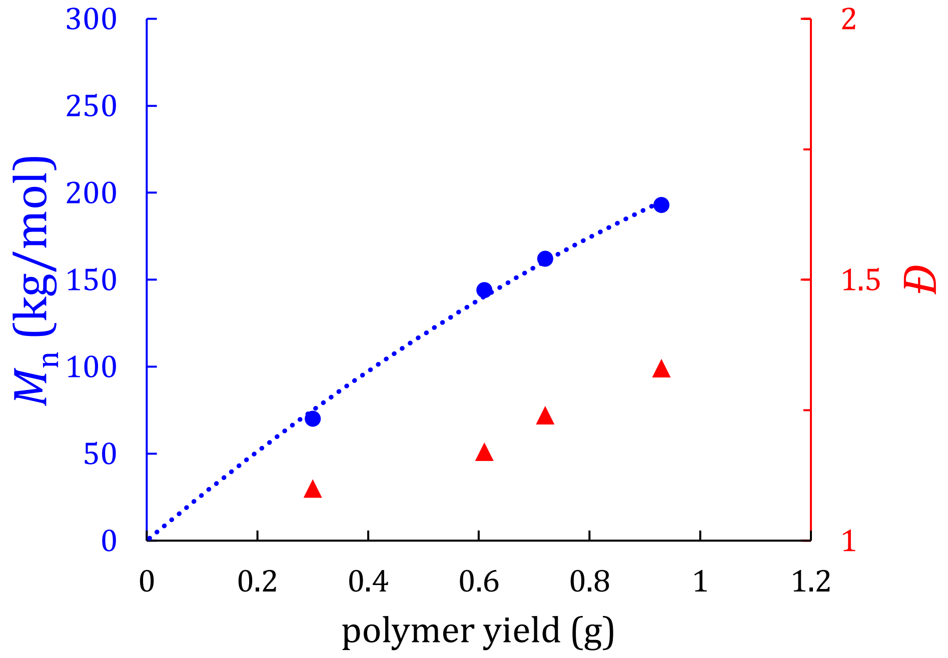

Figure S10. Plot of M_n (blue circles) and *Đ* (red triangles) as a function of polymer yield using **1**/PMAO-IP at 80 °C.

LB04225 Printed: Wednesday, December 21, 2016 10:35:41
C:\Users\Lauren\Desktop\gpc\2016-12-21_02:57:40_LB04225_01.vdt

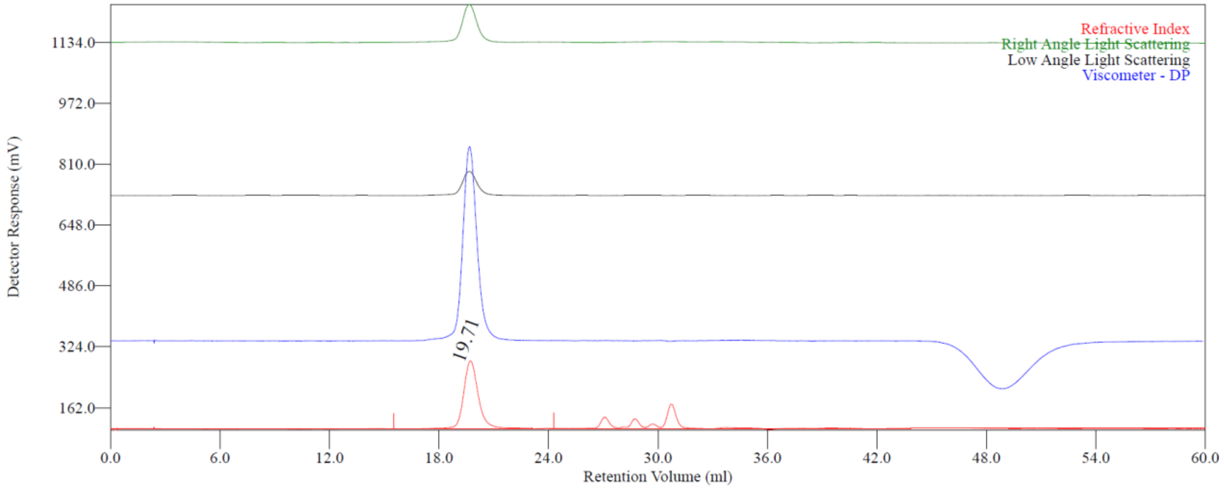

Molecular weight data for peak 1 is non-monotonic. Please read Help - FAQ entry.

Multi-Detectors - Homopolymers : Results

Peak RV - (ml)	19.707
Mn - (Daltons)	44,340
Mw - (Daltons)	59,631
Mz - (Daltons)	70,535
Mp - (Daltons)	56,965
Mw / Mn	1.345
Percent Above Mw: 0	100.000
Percent Below Mw: 0	0.000
IV - (dl/g)	1.1117
Rh(w) - (nm)	10.058
Rg(w) - (nm)	No Calc
Wt Fr (Peak)	1.000
Mark-Houwink a	0.698
Mark-Houwink logK	-3.277
Branches	0.000
Branch Freq.	0.000
RI Area - (mvml)	167.75
UV Area - (mvml)	0.00
RALS Area - (mvml)	97.10
LALS Area - (mvml)	62.50
IVDP Area - (mvml)	474.82

Sample Parameters	Input	Calculated
Sample Conc - (mg/ml)	3.800	3.288
Sample Recovery (%)	0.000	86.516
dn/dc - (ml/g)	0.1040	0.0000
dA/dc - (ml/g)	1.0000	0.0000

Annotation	
Method File	after RI intensity increase-0003.vcm
Limits File	
Date Acquired	Dec 21, 2016 - 02:57:40
Solvent	TCB
Acquisition Operator	admin : Administrator
Calculation Operator	admin : Administrator
Column Set	CLM6210 - HT X 3
System	System 1
Flow Rate - (ml/min)	1.000
Inj Volume - (ul)	200.0
Volume Increment - (ml)	0.00333
Detector Temp. - (deg C)	160.0
Column Temp. - (deg C)	160.0
OmniSEC Build Number	406

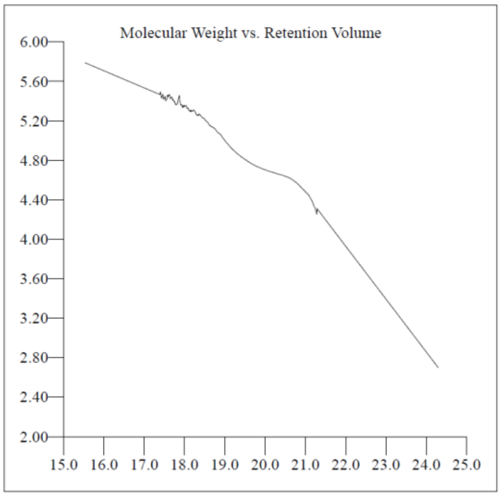

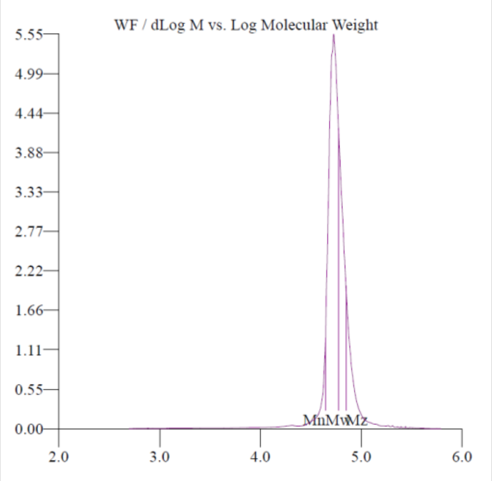

Figure S11. GPC of polyethylene. (Table 1, Entry 1)

LB04154 Printed: Thursday, December 15, 2016 11:46:29
C:\Users\Lauren\Desktop\gpc\2016-12-13_23;02;07_LB04154_01.vdt

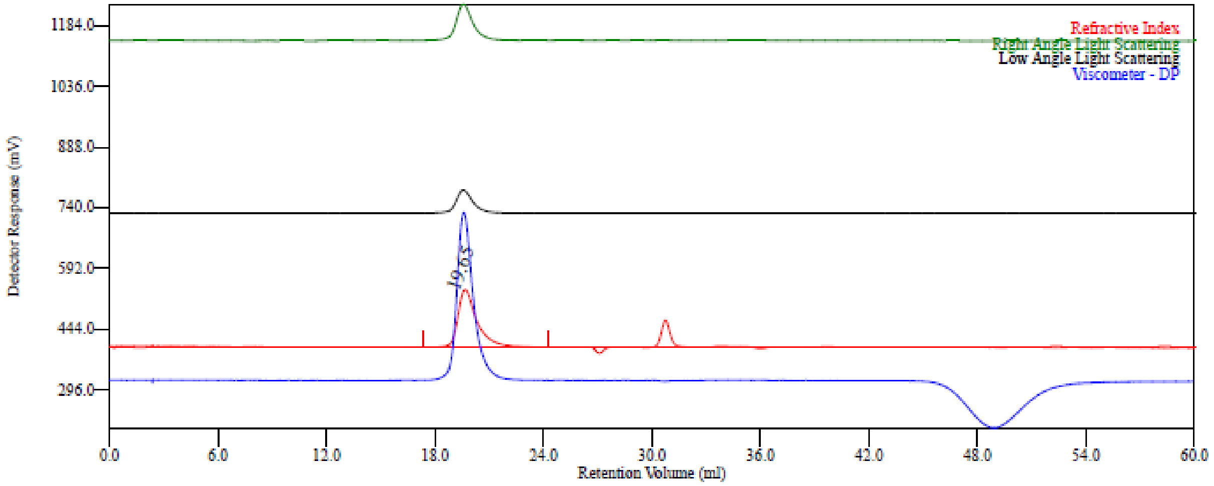

Multi-Detectors - Homopolymers : Results

Peak RV - (ml)	19.653
Mn - (Daltons)	49,456
Mw - (Daltons)	60,436
Mz - (Daltons)	76,062
Mp - (Daltons)	65,822
Mw / Mn	1.222
Percent Above Mw: 0	100.000
Percent Below Mw: 0	0.000
IV - (dl/g)	1.0271
Rh(w) - (nm)	9.771
Rg(w) - (nm)	21.890
Wt Fr (Peak)	1.000
Mark-Houwink a	0.745
Mark-Houwink logK	-3.539
Branches	0.000
Branch Freq.	0.000
RI Area - (mvml)	171.13
UV Area - (mvml)	0.00
RALS Area - (mvml)	97.55
LALS Area - (mvml)	61.80
IVDP Area - (mvml)	446.55

Sample Parameters	Input	Calculated
Sample Conc - (mg/ml)	4.230	3.354
Sample Recovery (%)	0.000	79.284
dn/dc - (ml/g)	0.1040	0.0000
dA/dc - (ml/g)	1.0000	0.0000

Annotation	
Method File	after RI intensity increase-0003.vcm
Limits File	
Date Acquired	Dec 13, 2016 - 23:02:07
Solvent	TCB
Acquisition Operator	admin : Administrator
Calculation Operator	admin : Administrator
Column Set	CLM6210 - HT X 3
System	System 1
Flow Rate - (ml/min)	1.000
Inj Volume - (ul)	200.0
Volume Increment - (ml)	0.00333
Detector Temp. - (deg C)	160.0
Column Temp. - (deg C)	160.0
OmniSEC Build Number	406

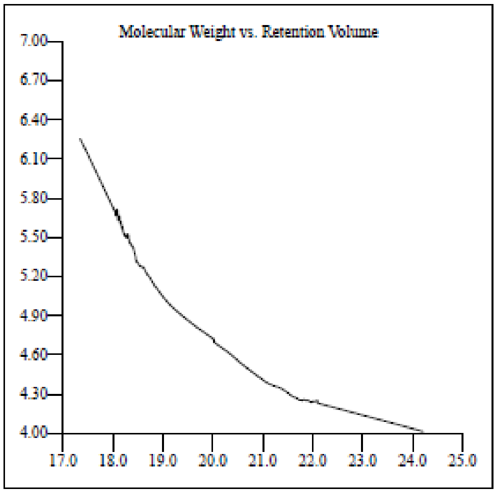

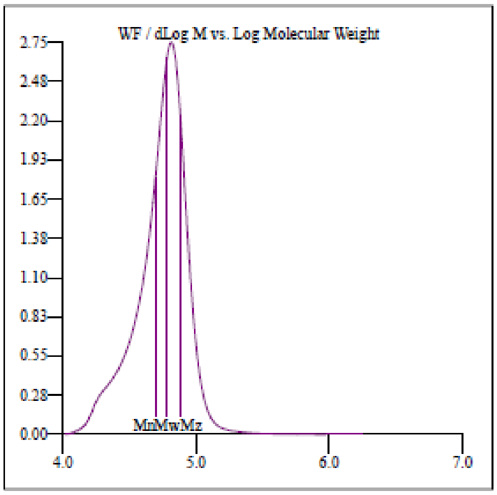

Figure S12. GPC of polyethylene. (Table 1, Entry 2)

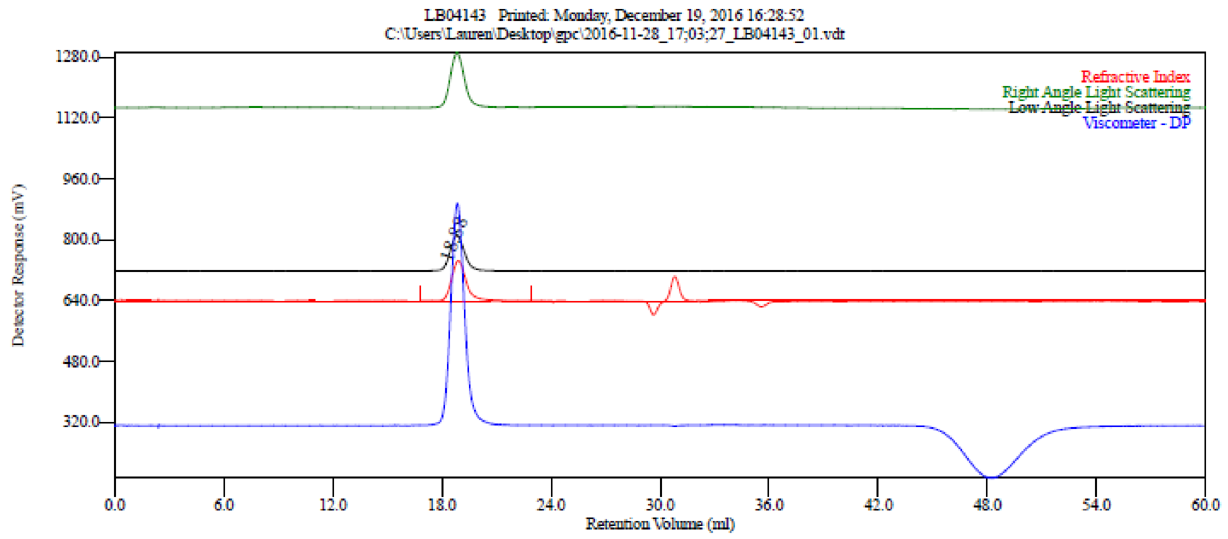

Multi-Detectors - Homopolymers : Results

Peak RV - (ml)	18.883
Mn - (Daltons)	128,197
Mw - (Daltons)	146,658
Mz - (Daltons)	158,072
Mp - (Daltons)	148,166
Mw / Mn	1.144
Percent Above Mw: 0	100.000
Percent Below Mw: 0	0.000
IV - (dl/g)	2.0571
Rh(w) - (nm)	16.703
Rg(w) - (nm)	24.630
Wt Fr (Peak)	1.000
Mark-Houwink a	0.770
Mark-Houwink logK	-3.660
Branches	0.000
Branch Freq.	0.000
RI Area - (mvml)	98.63
UV Area - (mvml)	0.00
RALS Area - (mvml)	130.80
LALS Area - (mvml)	85.92
IVDP Area - (mvml)	516.59

Sample Parameters	Input	Calculated
Sample Conc - (mg/ml)	2.450	1.933
Sample Recovery (%)	0.000	78.895
dn/dc - (ml/g)	0.1040	0.0000
dA/dc - (ml/g)	1.0000	0.0000

Annotation	
Method File	after RI intensity increase-0003.vcm
Limits File	
Date Acquired	Nov 28, 2016 - 17:03:27
Solvent	TCB
Acquisition Operator	admin : Administrator
Calculation Operator	admin : Administrator
Column Set	CLM6210 - HT X 3
System	System 1
Flow Rate - (ml/min)	1.000
Inj Volume - (ul)	200.0
Volume Increment - (ml)	0.00333
Detector Temp. - (deg C)	160.0
Column Temp. - (deg C)	160.0
OmniSEC Build Number	406

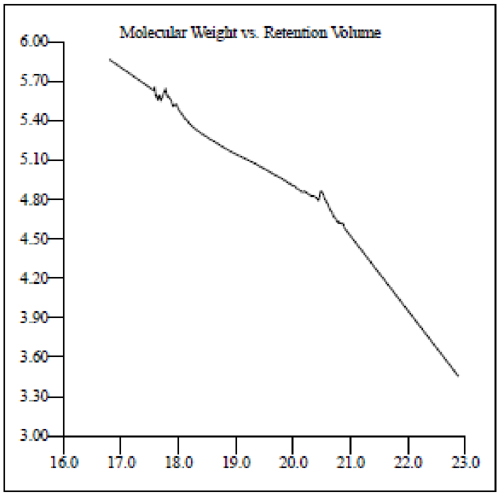

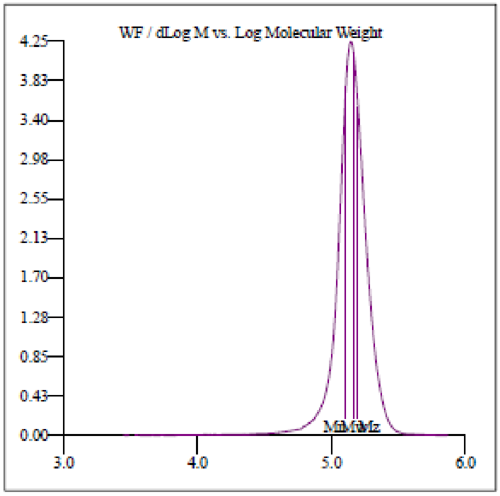

Figure S13. GPC of polyethylene. (Table 1, Entry 3)

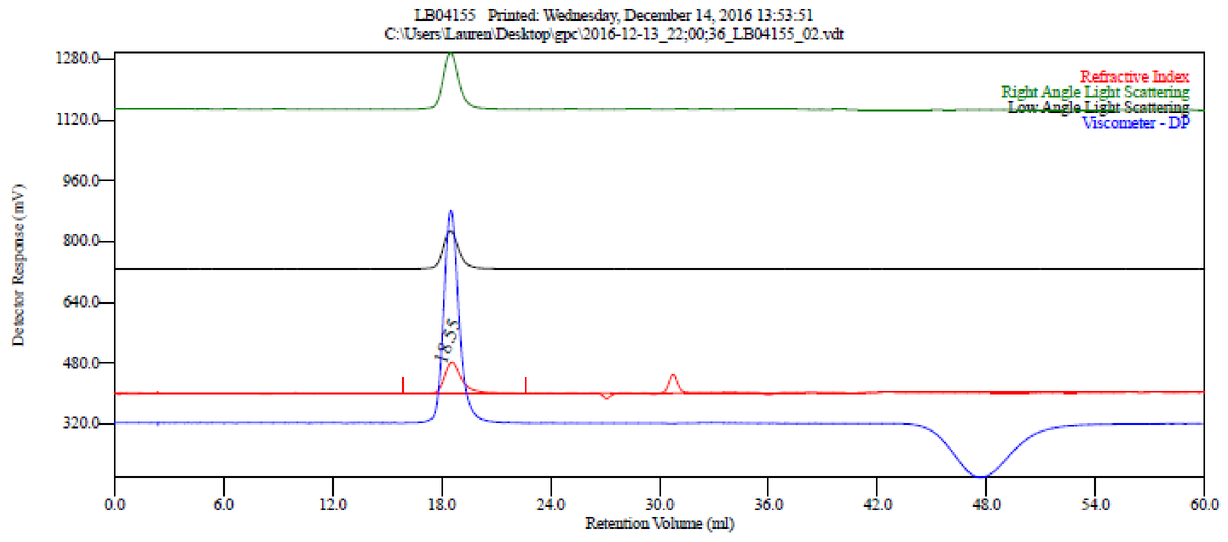

Multi-Detectors - Homopolymers : Results

Peak RV - (ml)	18.550
Mn - (Daltons)	163,983
Mw - (Daltons)	192,785
Mz - (Daltons)	244,962
Mp - (Daltons)	202,667
Mw / Mn	1.176
Percent Above Mw: 0	100.000
Percent Below Mw: 0	0.000
IV - (dl/g)	2.4351
Rh(w) - (nm)	19.241
Rg(w) - (nm)	28.722
Wt Fr (Peak)	1.000
Mark-Houwink a	0.781
Mark-Houwink logK	-3.731
Branches	0.000
Branch Freq.	0.000
RI Area - (mvml)	88.80
UV Area - (mvml)	0.00
RALS Area - (mvml)	149.88
LALS Area - (mvml)	100.87
IVDP Area - (mvml)	550.33

Sample Parameters	Input	Calculated
Sample Conc - (mg/ml)	2.010	1.740
Sample Recovery (%)	0.000	86.584
dn/dc - (ml/g)	0.1040	0.0000
dA/dc - (ml/g)	1.0000	0.0000

Annotation	
Method File	after RI intensity increase-0003.vcm
Limits File	
Date Acquired	Dec 13, 2016 - 22:00:36
Solvent	TCB
Acquisition Operator	admin : Administrator
Calculation Operator	admin : Administrator
Column Set	CLM6210 - HT X 3
System	System 1
Flow Rate - (ml/min)	1.000
Inj Volume - (ul)	200.0
Volume Increment - (ml)	0.00333
Detector Temp. - (deg C)	160.0
Column Temp. - (deg C)	160.0
OmniSEC Build Number	406

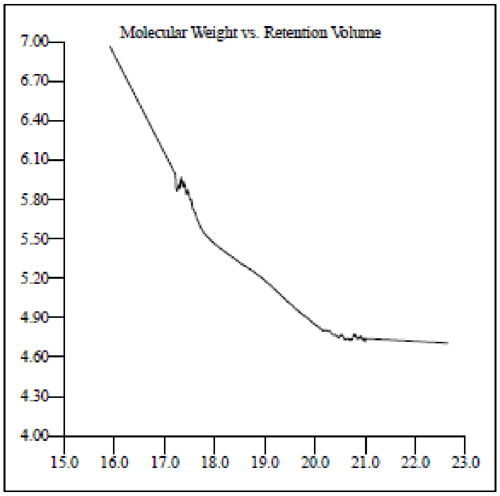

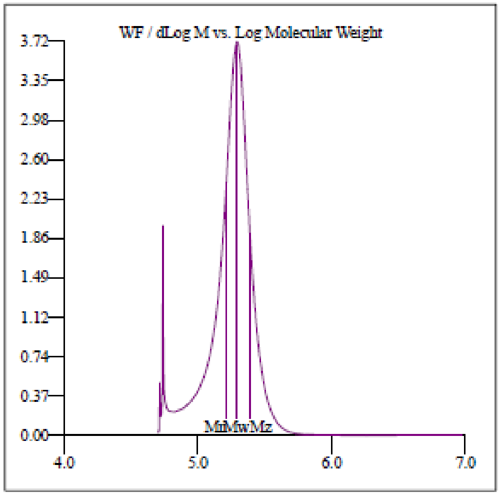

Figure S14. GPC of polyethylene. (Table 1, Entry 4)

LB04150 Printed: Tuesday, December 13, 2016 12:41:31
C:\Users\Lauren\Desktop\gpc\2016-11-28_19;06;29_LB04150_01.vdt

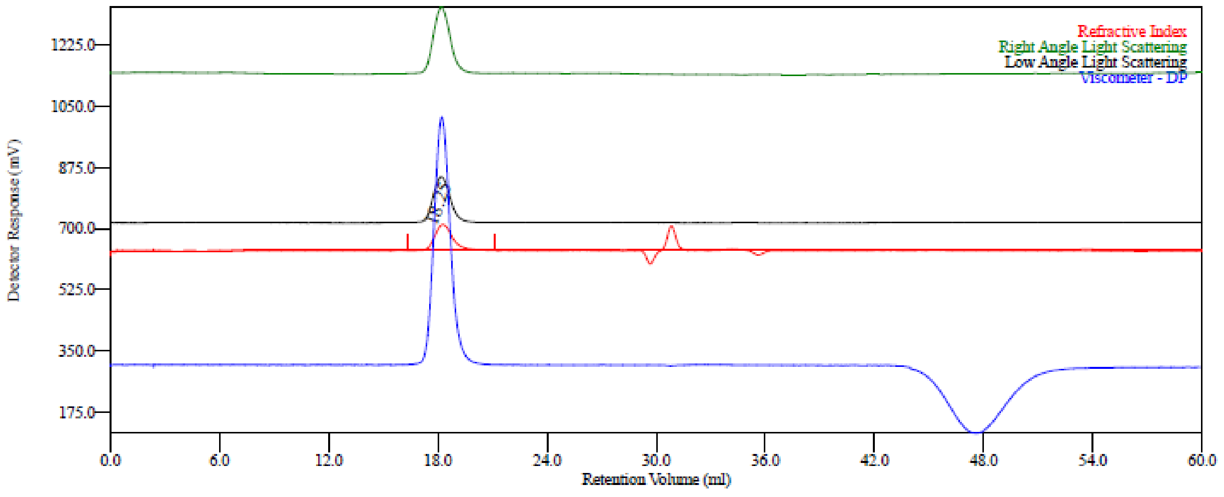

Multi-Detectors - Homopolymers : Results

Peak RV - (ml)	18.250
Mn - (Daltons)	244,076
Mw - (Daltons)	285,631
Mz - (Daltons)	312,428
Mp - (Daltons)	296,670
Mw / Mn	1.170
Percent Above Mw: 0	100.000
Percent Below Mw: 0	0.000
IV - (dl/g)	3.4779
Rh(w) - (nm)	24.816
Rg(w) - (nm)	33.330
Wt Fr (Peak)	1.000
Mark-Houwink a	0.797
Mark-Houwink logK	-3.806
Branches	0.000
Branch Freq.	0.000
RI Area - (mvml)	82.65
UV Area - (mvml)	0.00
RALS Area - (mvml)	201.92
LALS Area - (mvml)	140.56
IVDP Area - (mvml)	739.40

Sample Parameters	Input	Calculated
Sample Conc - (mg/ml)	2.220	1.620
Sample Recovery (%)	0.000	72.964
dn/dc - (ml/g)	0.1040	0.0000
dA/dc - (ml/g)	1.0000	0.0000

Annotation	
Method File	after RI intensity increase-0003.vcm
Limits File	
Date Acquired	Nov 28, 2016 - 19:06:29
Solvent	TCB
Acquisition Operator	admin : Administrator
Calculation Operator	admin : Administrator
Column Set	CLM6210 - HT X 3
System	System 1
Flow Rate - (ml/min)	1.000
Inj Volume - (ul)	200.0
Volume Increment - (ml)	0.00333
Detector Temp. - (deg C)	160.0
Column Temp. - (deg C)	160.0
OmniSEC Build Number	406

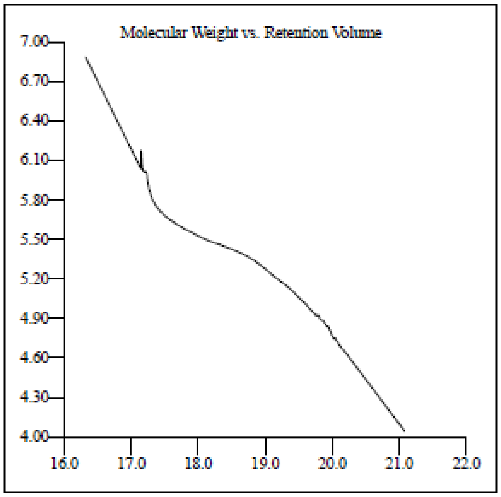

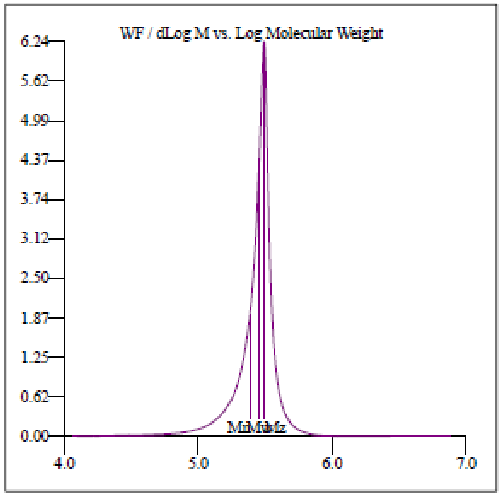

Figure S15. GPC of polyethylene. (Table 1, Entry 5)

LB04224-2 Printed: Wednesday, December 21, 2016 10:01:40
C:\Users\Lauren\Desktop\gpc\2016-12-21_03;59;09_LB04224-2_01.vdt

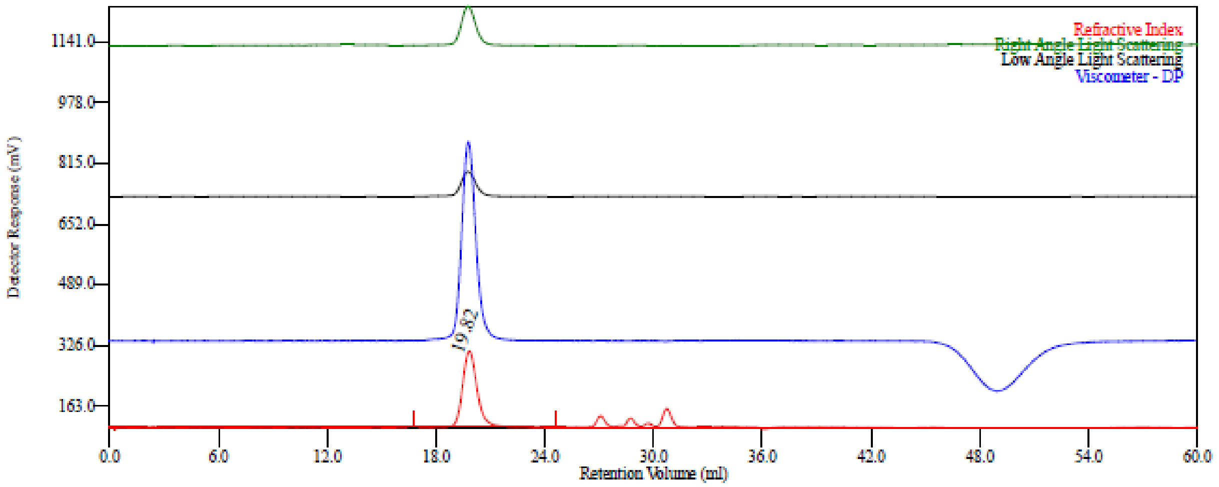

Multi-Detectors - Homopolymers : Results

Peak RV - (ml)	19.817
Mn - (Daltons)	40,284
Mw - (Daltons)	55,187
Mz - (Daltons)	63,085
Mp - (Daltons)	55,011
Mw / Mn	1.370
Percent Above Mw: 0	100.000
Percent Below Mw: 0	0.000
IV - (dl/g)	1.0062
Rh(w) - (nm)	9.482
Rg(w) - (nm)	22.300
Wt Fr (Peak)	1.000
Mark-Houwink a	0.637
Mark-Houwink logK	-3.013
Branches	0.000
Branch Freq.	0.000
RI Area - (mvml)	192.56
UV Area - (mvml)	0.00
RALS Area - (mvml)	98.78
LALS Area - (mvml)	64.61
IVDP Area - (mvml)	492.46

Sample Parameters	Input	Calculated
Sample Conc - (mg/ml)	4.460	3.774
Sample Recovery (%)	0.000	84.613
dn/dc - (ml/g)	0.1040	0.0000
dA/dc - (ml/g)	1.0000	0.0000

Annotation	
Method File	after RI intensity increase-0003.vcm
Limits File	
Date Acquired	Dec 21, 2016 - 03:59:09
Solvent	TCB
Acquisition Operator	admin : Administrator
Calculation Operator	admin : Administrator
Column Set	CLM6210 - HT X 3
System	System 1
Flow Rate - (ml/min)	1.000
Inj Volume - (ul)	200.0
Volume Increment - (ml)	0.00333
Detector Temp. - (deg C)	160.0
Column Temp. - (deg C)	160.0
OmniSEC Build Number	406

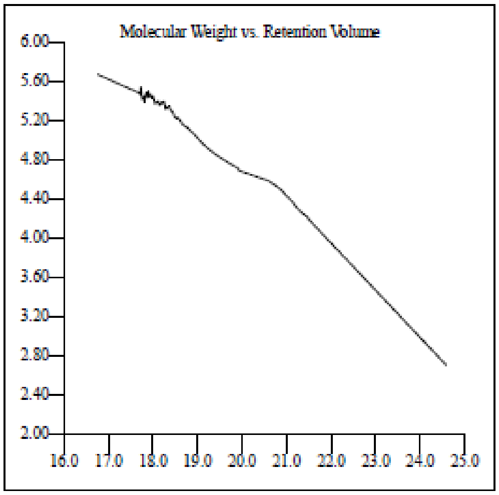

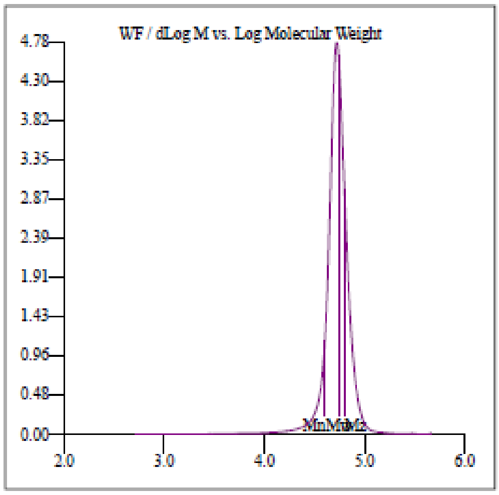

Figure S16. GPC of polyethylene. (Table 1, Entry 6)

LB04156 Printed: Wednesday, December 14, 2016 13:49:10
C:\Users\Lauren\Desktop\gpc\2016-12-14_02;06;38_LB04156_02.vdt

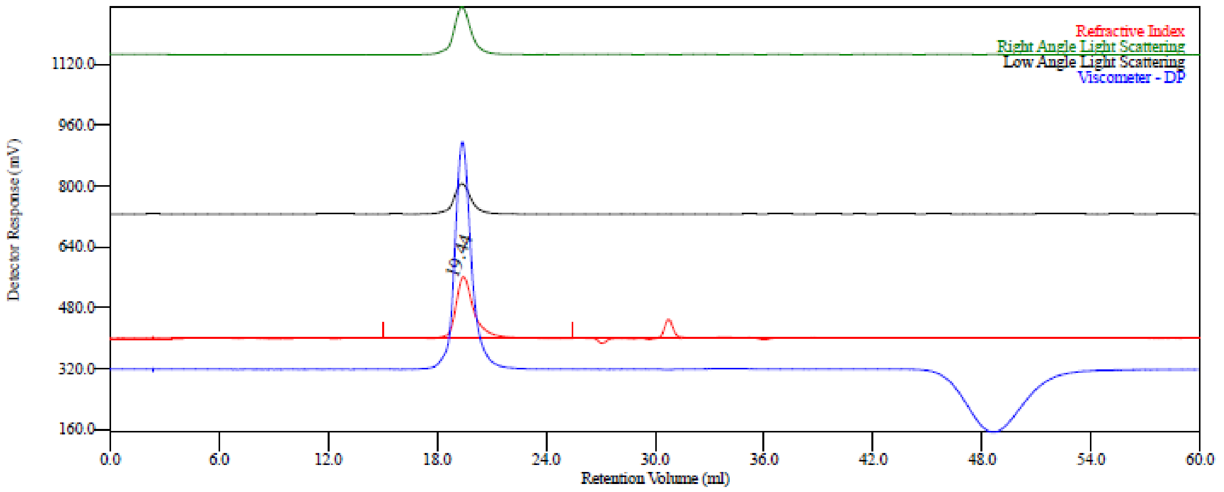

Multi-Detectors - Homopolymers : Results

Peak RV - (ml)	19.443
Mn - (Daltons)	67,160
Mw - (Daltons)	80,607
Mz - (Daltons)	99,680
Mp - (Daltons)	82,091
Mw / Mn	1.200
Percent Above Mw: 0	100.000
Percent Below Mw: 0	0.000
IV - (dl/g)	1.3366
Rh(w) - (nm)	11.768
Rg(w) - (nm)	23.754
Wt Fr (Peak)	1.000
Mark-Houwink a	0.703
Mark-Houwink logK	-3.316
Branches	0.000
Branch Freq.	0.000
RI Area - (mvml)	187.79
UV Area - (mvml)	0.00
RALS Area - (mvml)	139.72
LALS Area - (mvml)	90.81
IVDP Area - (mvml)	640.91

Sample Parameters	Input	Calculated
Sample Conc - (mg/ml)	4.400	3.680
Sample Recovery (%)	0.000	83.644
dn/dc - (ml/g)	0.1040	0.0000
dA/dc - (ml/g)	1.0000	0.0000

Annotation	
Method File	after RI intensity increase-0003.vcm
Limits File	
Date Acquired	Dec 14, 2016 - 02:06:38
Solvent	TCB
Acquisition Operator	admin : Administrator
Calculation Operator	admin : Administrator
Column Set	CLM6210 - HT X 3
System	System 1
Flow Rate - (ml/min)	1.000
Inj Volume - (ul)	200.0
Volume Increment - (ml)	0.00333
Detector Temp. - (deg C)	160.0
Column Temp. - (deg C)	160.0
OmniSEC Build Number	406

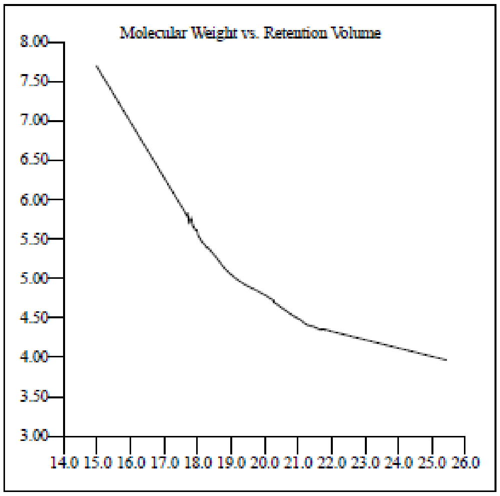

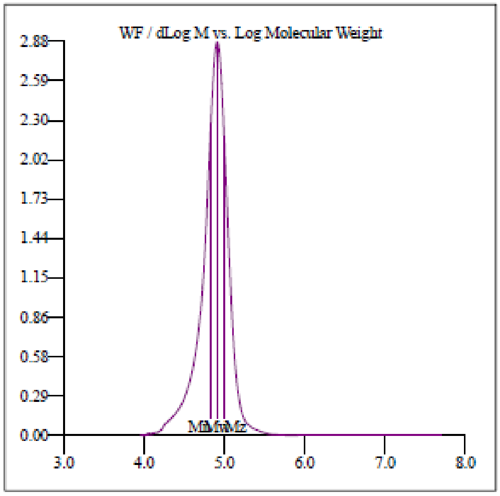

Figure S17. GPC of polyethylene. (Table 1, Entry 7)

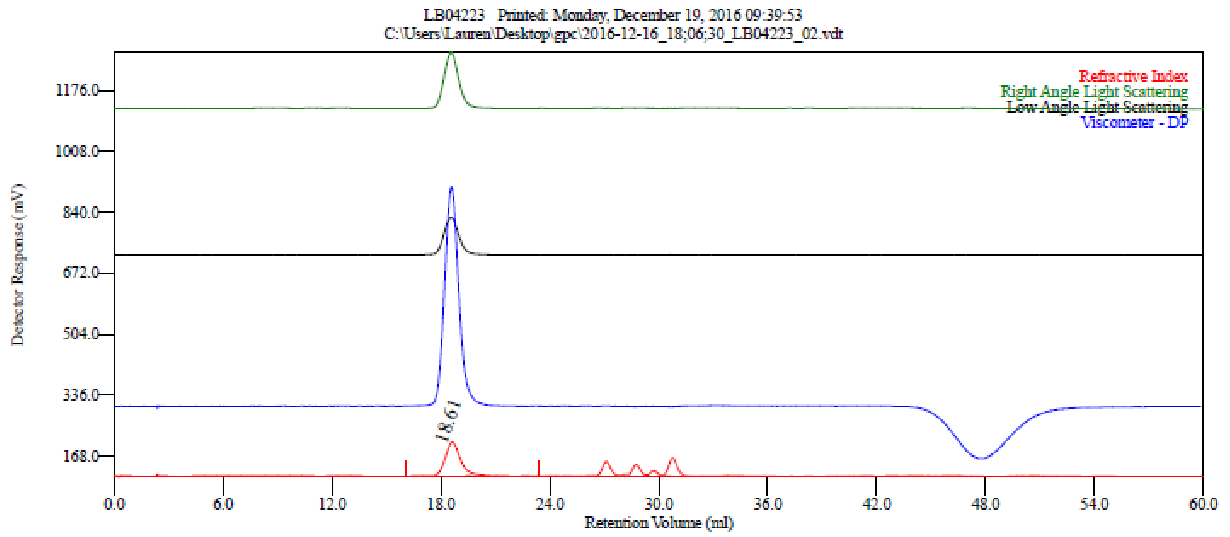

Molecular weight data for peak 1 is non-monotonic. Please read Help - FAQ entry.

Multi-Detectors - Homopolymers : Results

Peak RV - (ml)	18.613
Mn - (Daltons)	154,545
Mw - (Daltons)	181,453
Mz - (Daltons)	195,478
Mp - (Daltons)	187,728
Mw / Mn	1.174
Percent Above Mw: 0	100.000
Percent Below Mw: 0	0.000
IV - (dl/g)	2.3853
Rh(w) - (nm)	18.832
Rg(w) - (nm)	34.274
Wt Fr (Peak)	1.000
Mark-Houwink a	0.768
Mark-Houwink logK	-3.658
Branches	0.000
Branch Freq.	0.000
RI Area - (mvml)	91.69
UV Area - (mvml)	0.00
RALS Area - (mvml)	144.76
LALS Area - (mvml)	98.39
IVDP Area - (mvml)	559.66

Sample Parameters	Input	Calculated
Sample Conc - (mg/ml)	1.980	1.797
Sample Recovery (%)	0.000	90.754
dn/dc - (ml/g)	0.1040	0.0000
dA/dc - (ml/g)	1.0000	0.0000

Annotation	
Method File	after RI intensity increase-0003.vcm
Limits File	
Date Acquired	Dec 16, 2016 - 18:06:30
Solvent	TCB
Acquisition Operator	admin : Administrator
Calculation Operator	admin : Administrator
Column Set	CLM6210 - HT X 3
System	System 1
Flow Rate - (ml/min)	1.000
Inj Volume - (ul)	200.0
Volume Increment - (ml)	0.00333
Detector Temp. - (deg C)	160.0
Column Temp. - (deg C)	160.0
OmniSEC Build Number	406

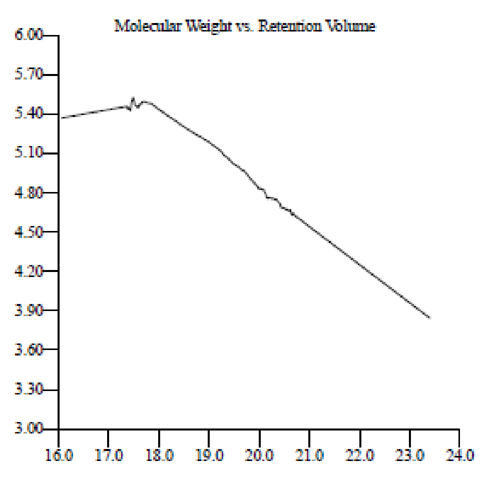

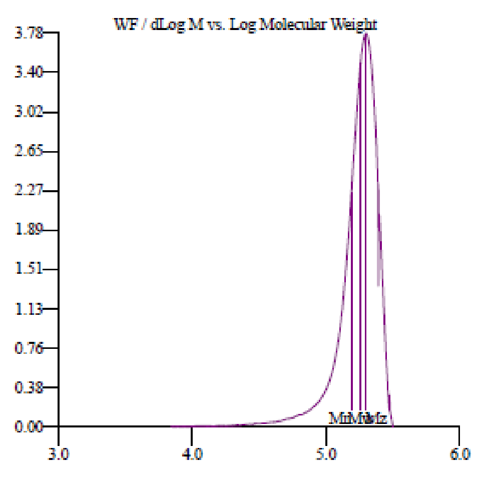

Figure S18. GPC of polyethylene. (Table 1, Entry 8)

LB04157 Printed: Tuesday, December 13, 2016 12:44:44
C:\Users\Lauren\Desktop\gpc\2016-11-29_00;14;04_LB04157_01.vdt

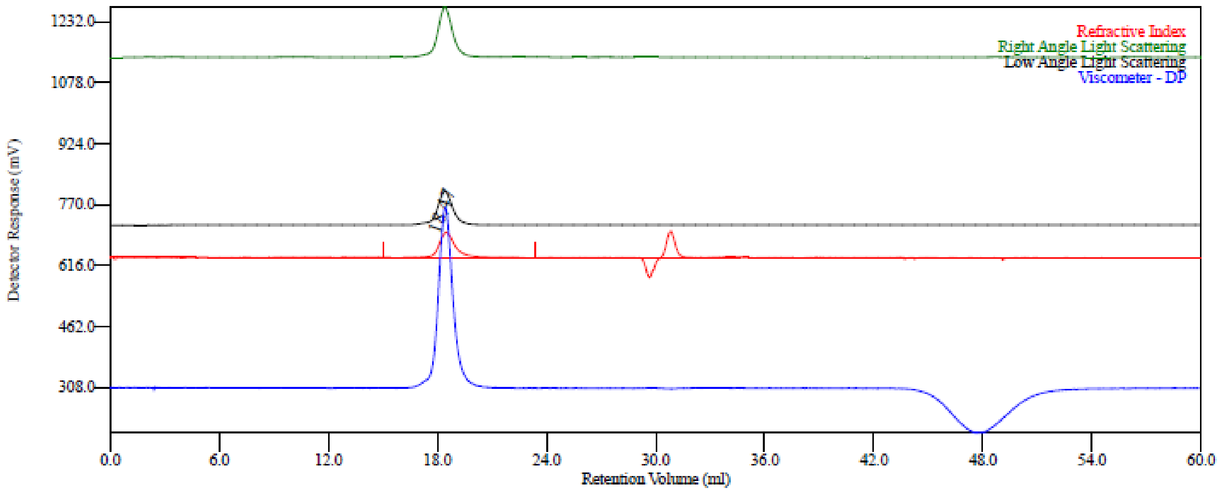

Multi-Detectors - Homopolymers : Results

Peak RV - (ml)	18.470
Mn - (Daltons)	191,059
Mw - (Daltons)	217,559
Mz - (Daltons)	245,481
Mp - (Daltons)	223,227
Mw / Mn	1.139
Percent Above Mw: 0	100.000
Percent Below Mw: 0	0.000
IV - (dl/g)	2.6599
Rh(w) - (nm)	20.696
Rg(w) - (nm)	30.512
Wt Fr (Peak)	1.000
Mark-Houwink a	0.682
Mark-Houwink logK	-3.214
Branches	0.000
Branch Freq.	0.000
RI Area - (mvml)	63.78
UV Area - (mvml)	0.00
RALS Area - (mvml)	121.08
LALS Area - (mvml)	81.61
IVDP Area - (mvml)	427.16

Sample Parameters	Input	Calculated
Sample Conc - (mg/ml)	2.070	1.250
Sample Recovery (%)	0.000	60.382
dn/dc - (ml/g)	0.1040	0.0000
dA/dc - (ml/g)	1.0000	0.0000

Annotation	
Method File	after RI intensity increase-0003.vcm
Limits File	
Date Acquired	Nov 29, 2016 - 00:14:04
Solvent	TCB
Acquisition Operator	admin : Administrator
Calculation Operator	admin : Administrator
Column Set	CLM6210 - HT X 3
System	System 1
Flow Rate - (ml/min)	1.000
Inj Volume - (ul)	200.0
Volume Increment - (ml)	0.00333
Detector Temp. - (deg C)	160.0
Column Temp. - (deg C)	160.0
OmniSEC Build Number	406

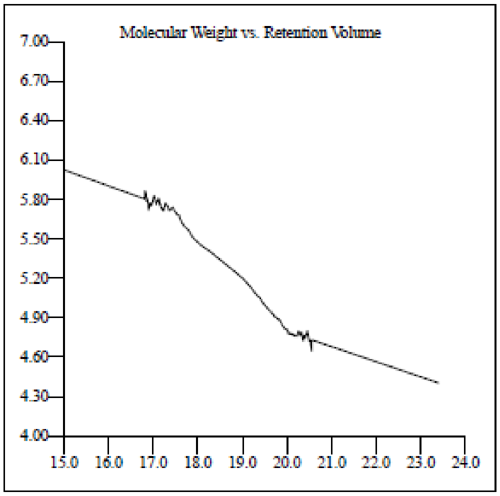

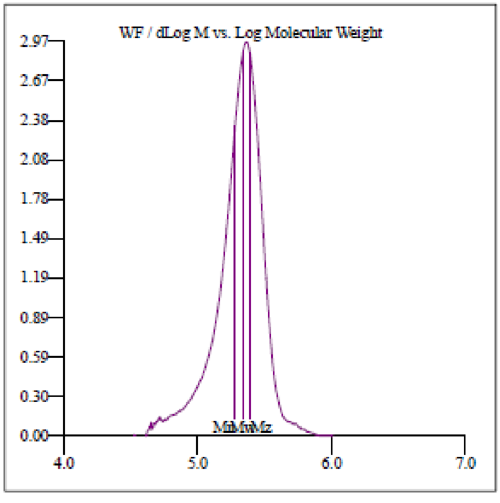

Figure S19. GPC of polyethylene. (Table 1, Entry 9)

LB04151 Printed: Wednesday, December 21, 2016 10:46:58
C:\Users\Lauren\Desktop\gpc\2016-12-13_00;29;37_LB04151_01.vdt

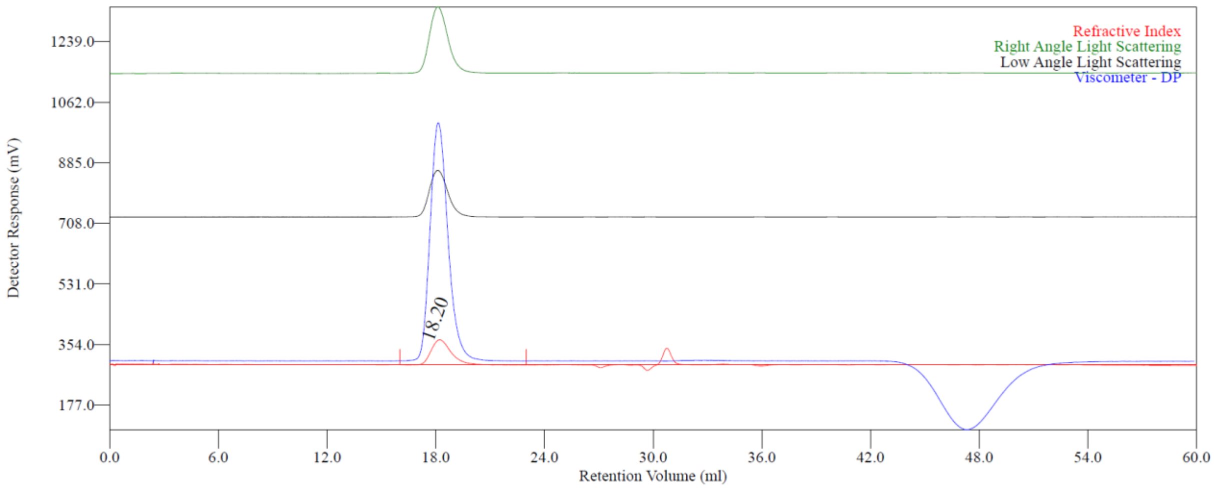

Molecular weight data for peak 1 is non-monotonic. Please read Help - FAQ entry.

Multi-Detectors - Homopolymers : Results

Peak RV - (ml)	18.203
Mn - (Daltons)	253,440
Mw - (Daltons)	290,065
Mz - (Daltons)	321,609
Mp - (Daltons)	308,691
Mw / Mn	1.145
Percent Above Mw: 0	100.000
Percent Below Mw: 0	0.000
IV - (dl/g)	3.3094
Rh(w) - (nm)	24.498
Rg(w) - (nm)	34.271
Wt Fr (Peak)	1.000
Mark-Houwink a	0.851
Mark-Houwink logK	-4.123
Branches	0.000
Branch Freq.	0.000
RI Area - (mvml)	93.52
UV Area - (mvml)	0.00
RALS Area - (mvml)	229.84
LALS Area - (mvml)	162.33
IVDP Area - (mvml)	796.62

Sample Parameters	Input	Calculated
Sample Conc - (mg/ml)	2.320	1.833
Sample Recovery (%)	0.000	79.002
dn/dc - (ml/g)	0.1040	0.0000
dA/dc - (ml/g)	1.0000	0.0000

Annotation	
Method File	after RI intensity increase-0003.vcm
Limits File	
Date Acquired	Dec 13, 2016 - 00:29:37
Solvent	TCB
Acquisition Operator	admin : Administrator
Calculation Operator	admin : Administrator
Column Set	CLM6210 - HT X 3
System	System 1
Flow Rate - (ml/min)	1.000
Inj Volume - (ul)	200.0
Volume Increment - (ml)	0.00333
Detector Temp. - (deg C)	160.0
Column Temp. - (deg C)	160.0
OmniSEC Build Number	406

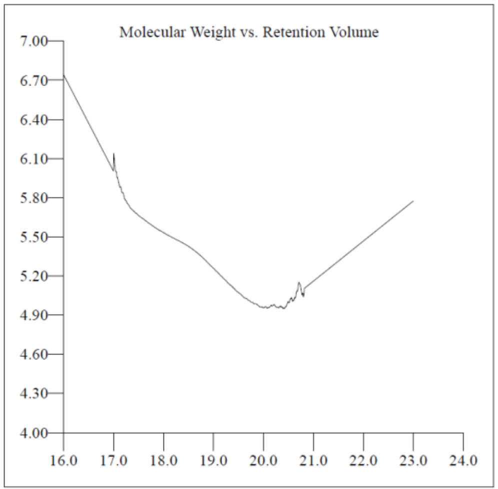

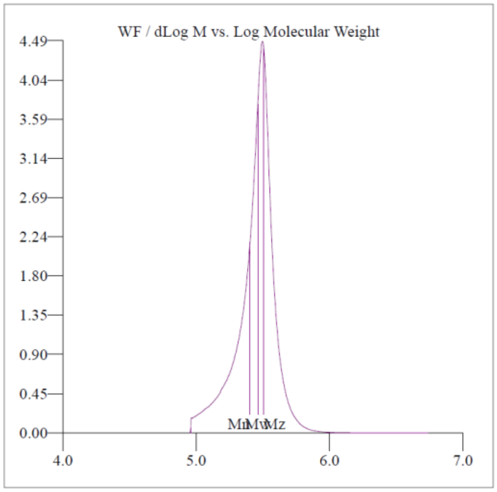

Figure S20. GPC of polyethylene. (Table 1, Entry 10)

LB04228 Printed: Tuesday, January 24, 2017 12:56:56
C:\Users\Lauren\Desktop\gpc\2017-01-23_15;22;10_LB04228_01.vdt

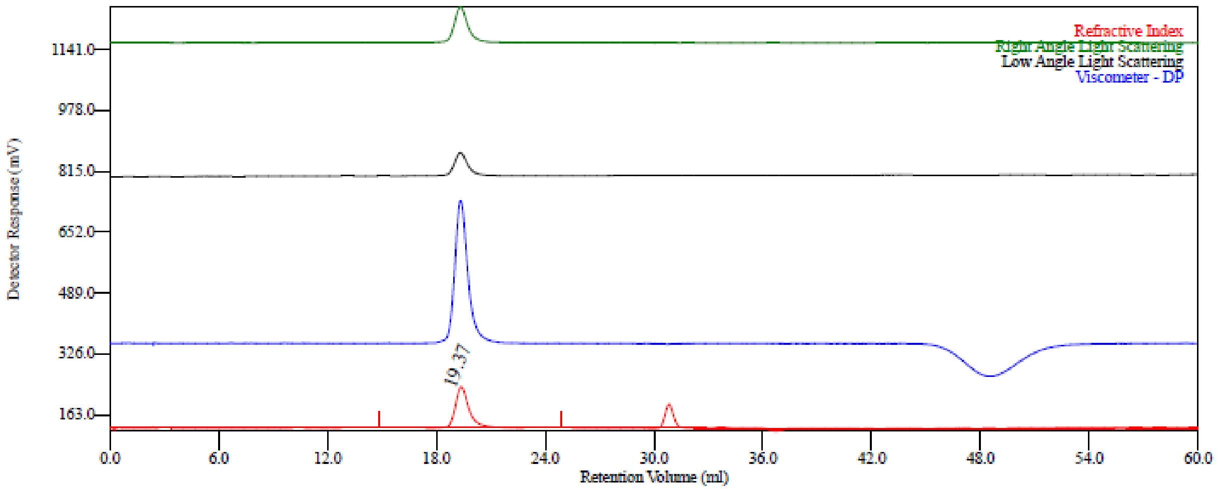

Multi-Detectors - Homopolymers : Results

Peak RV - (ml)	19.367
Mn - (Daltons)	69,570
Mw - (Daltons)	76,312
Mz - (Daltons)	87,100
Mp - (Daltons)	76,598
Mw / Mn	1.097
Percent Above Mw: 0	100.000
Percent Below Mw: 0	0.000
IV - (dl/g)	1.1574
Rh(w) - (nm)	10.981
Rg(w) - (nm)	No Calc
Wt Fr (Peak)	1.000
Mark-Houwink a	0.586
Mark-Houwink logK	-2.798
Branches	0.000
Branch Freq.	0.000
RI Area - (mvml)	97.98
UV Area - (mvml)	0.00
RALS Area - (mvml)	84.12
LALS Area - (mvml)	55.53
IVDP Area - (mvml)	329.60

Sample Parameters	Input	Calculated
Sample Conc - (mg/ml)	2.300	0.000
Sample Recovery (%)	0.000	83.720
dn/dc - (ml/g)	0.1040	0.0000
dA/dc - (ml/g)	1.0000	0.0000

Annotation	
Method File	Long Group 1-9-16-0000.vcm
Limits File	
Date Acquired	Jan 23, 2017 - 15:22:10
Solvent	TCB
Acquisition Operator	admin : Administrator
Calculation Operator	admin : Administrator
Column Set	CLM6210 - HT X 3
System	System 1
Flow Rate - (ml/min)	1.000
Inj Volume - (ul)	200.0
Volume Increment - (ml)	0.00333
Detector Temp. - (deg C)	160.0
Column Temp. - (deg C)	160.0
OmniSEC Build Number	406

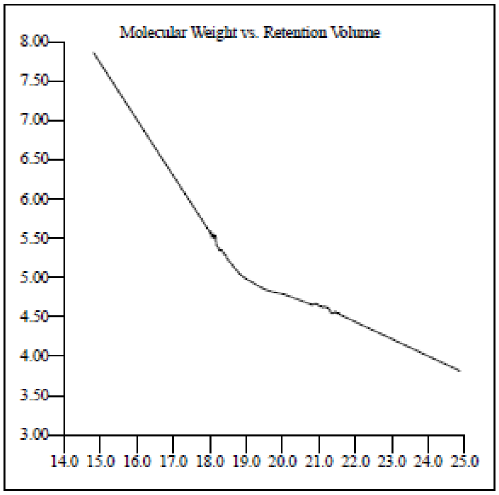

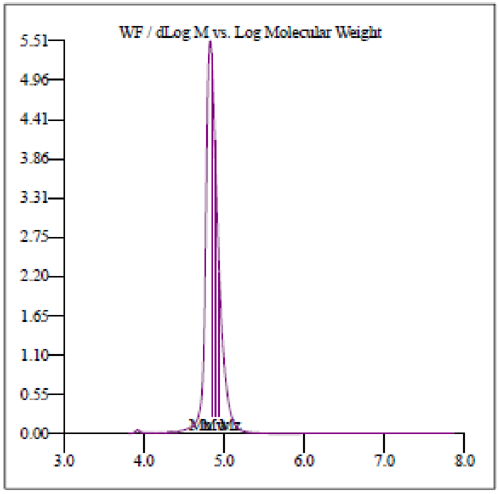

Figure S21. GPC of polyethylene. (Table 1, Entry 11)

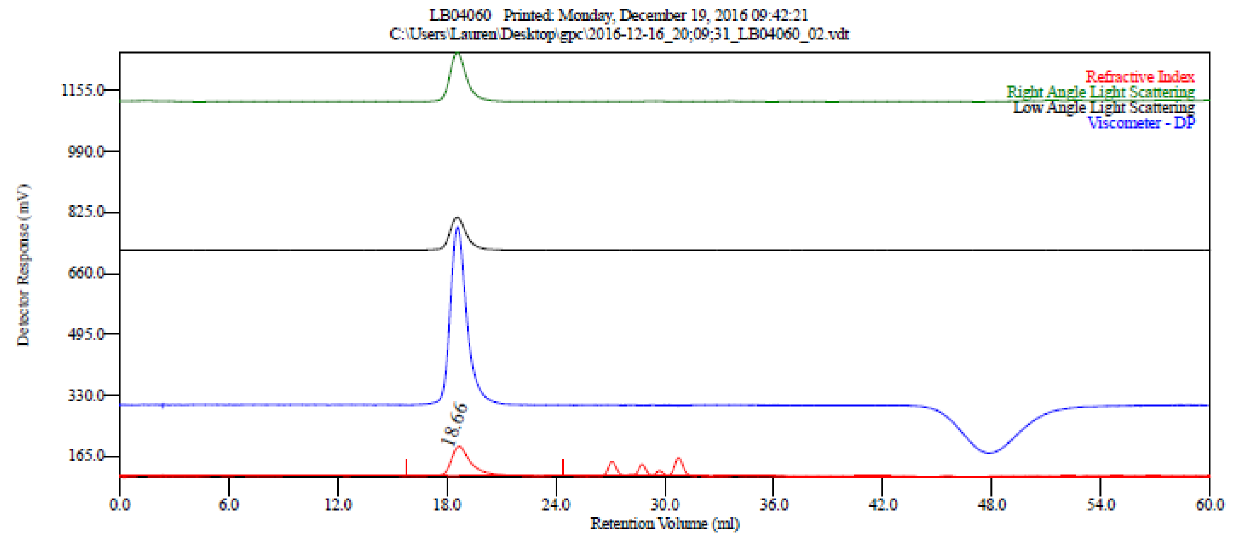

Multi-Detectors - Homopolymers : Results

Peak RV - (ml)	18.663
Mn - (Daltons)	113,544
Mw - (Daltons)	162,221
Mz - (Daltons)	192,266
Mp - (Daltons)	183,057
Mw / Mn	1.429
Percent Above Mw: 0	100.000
Percent Below Mw: 0	0.000
IV - (dl/g)	2.0336
Rh(w) - (nm)	17.003
Rg(w) - (nm)	29.339
Wt Fr (Peak)	1.000
Mark-Houwink a	0.814
Mark-Houwink logK	-3.921
Branches	0.000
Branch Freq.	0.000
RI Area - (mvml)	99.64
UV Area - (mvml)	0.00
RALS Area - (mvml)	140.15
LALS Area - (mvml)	96.05
IVDP Area - (mvml)	514.12

Sample Parameters	Input	Calculated
Sample Conc - (mg/ml)	2.050	1.953
Sample Recovery (%)	0.000	95.257
dn/dc - (ml/g)	0.1040	0.0000
dA/dc - (ml/g)	1.0000	0.0000

Annotation	
Method File	after RI intensity increase-0003.vcm
Limits File	
Date Acquired	Dec 16, 2016 - 20:09:31
Solvent	TCB
Acquisition Operator	admin : Administrator
Calculation Operator	admin : Administrator
Column Set	CLM6210 - HT X 3
System	System 1
Flow Rate - (ml/min)	1.000
Inj Volume - (ul)	200.0
Volume Increment - (ml)	0.00333
Detector Temp. - (deg C)	160.0
Column Temp. - (deg C)	160.0
OmniSEC Build Number	406

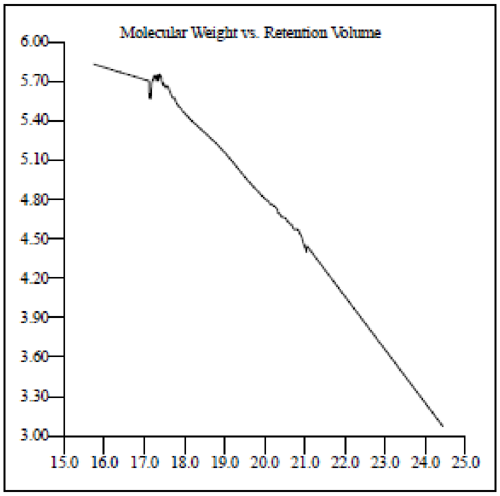

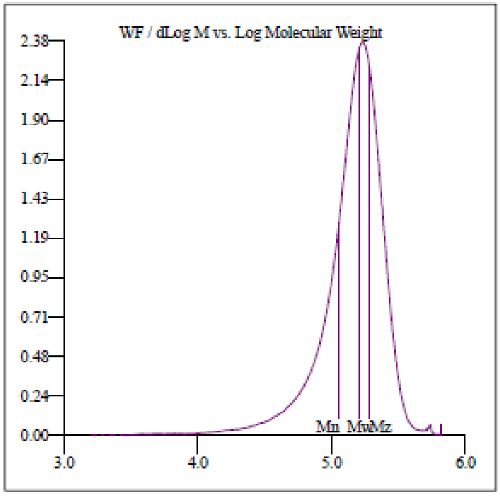

Figure S22. GPC of polyethylene. (Table 1, Entry 12)

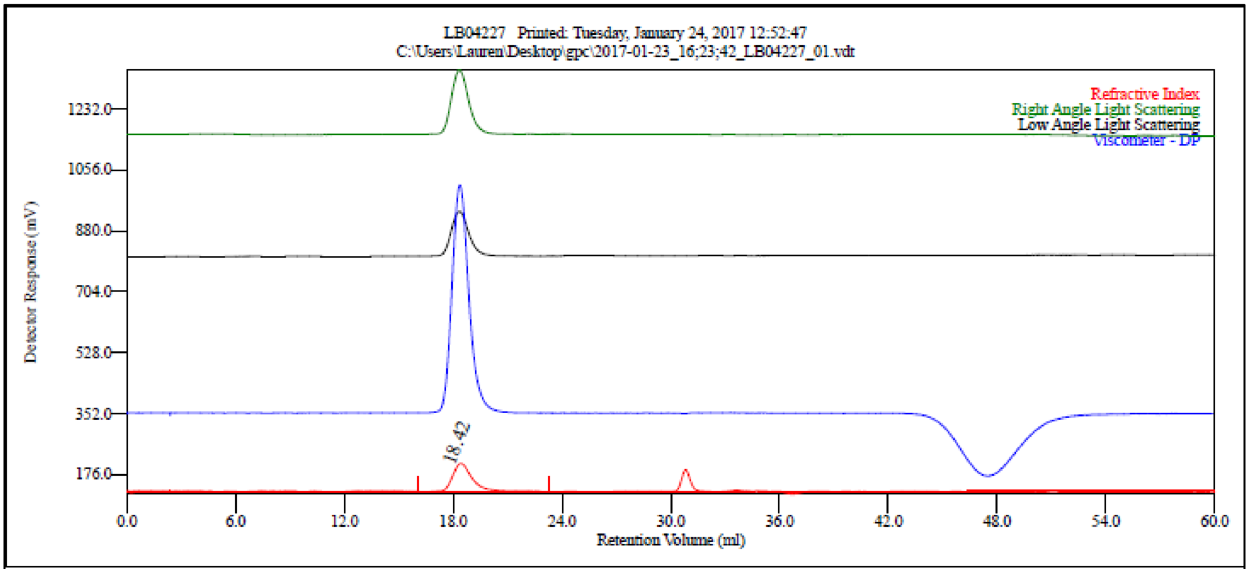

Multi-Detectors - Homopolymers : Results

Peak RV - (ml)	18.420
Mn - (Daltons)	161,556
Mw - (Daltons)	200,523
Mz - (Daltons)	223,178
Mp - (Daltons)	217,887
Mw / Mn	1.241
Percent Above Mw: 0	100.000
Percent Below Mw: 0	0.000
IV - (dl/g)	2.3981
Rh(w) - (nm)	19.417
Rg(w) - (nm)	27.824
Wt Fr (Peak)	1.000
Mark-Houwink a	0.806
Mark-Houwink logK	-3.884
Branches	0.000
Branch Freq.	0.000
RI Area - (mvml)	99.20
UV Area - (mvml)	0.00
RALS Area - (mvml)	205.48
LALS Area - (mvml)	145.34
IVDP Area - (mvml)	713.06

Sample Parameters	Input	Calculated
Sample Conc - (mg/ml)	2.310	0.000
Sample Recovery (%)	0.000	84.398
dn/dc - (ml/g)	0.1040	0.0000
dA/dc - (ml/g)	1.0000	0.0000

Annotation	
Method File	Long Group 1-9-16-0000.vcm
Limits File	
Date Acquired	Jan 23, 2017 - 16:23:42
Solvent	TCB
Acquisition Operator	admin : Administrator
Calculation Operator	admin : Administrator
Column Set	CLM6210 - HT X 3
System	System 1
Flow Rate - (ml/min)	1.000
Inj Volume - (ul)	200.0
Volume Increment - (ml)	0.00333
Detector Temp. - (deg C)	160.0
Column Temp. - (deg C)	160.0
OmniSEC Build Number	406

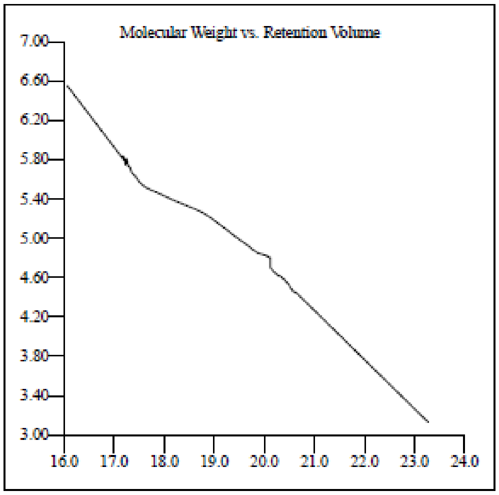

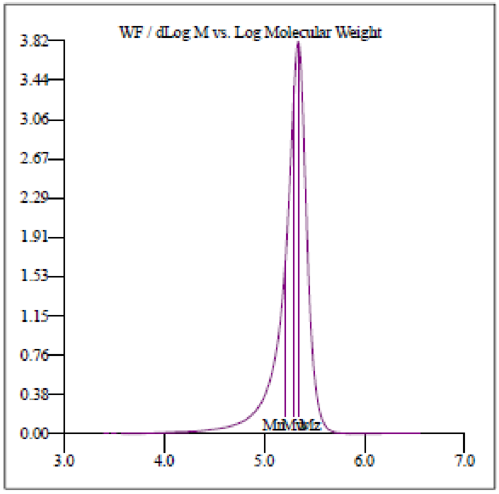

Figure S23. GPC of polyethylene. (Table 1, Entry 13)

LB04226-3 Printed: Tuesday, January 03, 2017 09:43:36
C:\Users\Lauren\Desktop\gpc\2017-01-02_19;16;37_LB04226-3_01.vdt

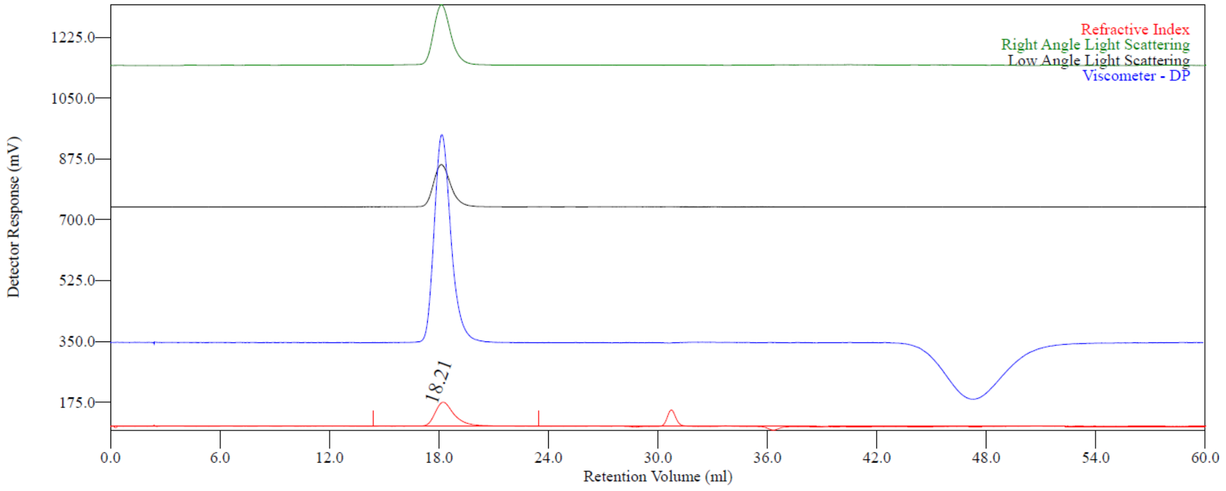

Multi-Detectors - Homopolymers : Results

Peak RV - (ml)	18.213
Mn - (Daltons)	192,793
Mw - (Daltons)	255,586
Mz - (Daltons)	292,681
Mp - (Daltons)	283,614
Mw / Mn	1.326
Percent Above Mw: 0	100.000
Percent Below Mw: 0	0.000
IV - (dl/g)	2.9555
Rh(w) - (nm)	22.488
Rg(w) - (nm)	No Calc
Wt Fr (Peak)	1.000
Mark-Houwink a	0.747
Mark-Houwink logK	-3.561
Branches	0.000
Branch Freq.	0.000
RI Area - (mvml)	85.46
UV Area - (mvml)	0.00
RALS Area - (mvml)	190.64
LALS Area - (mvml)	133.65
IVDP Area - (mvml)	643.32

Sample Parameters	Input	Calculated
Sample Conc - (mg/ml)	2.200	1.675
Sample Recovery (%)	0.000	76.133
dn/dc - (ml/g)	0.1040	0.0000
dA/dc - (ml/g)	1.0000	0.0000

Annotation	
Method File	after RI intensity increase-0003.vcm
Limits File	
Date Acquired	Jan 02, 2017 - 19:16:37
Solvent	TCB
Acquisition Operator	admin : Administrator
Calculation Operator	admin : Administrator
Column Set	CLM6210 - HT X 3
System	System 1
Flow Rate - (ml/min)	1.000
Inj Volume - (ul)	200.0
Volume Increment - (ml)	0.00333
Detector Temp. - (deg C)	160.0
Column Temp. - (deg C)	160.0
OmniSEC Build Number	406

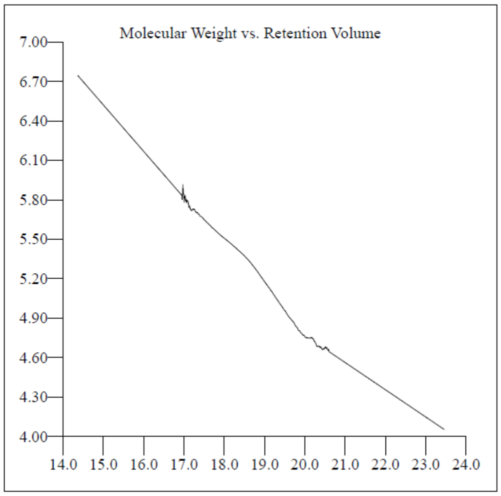

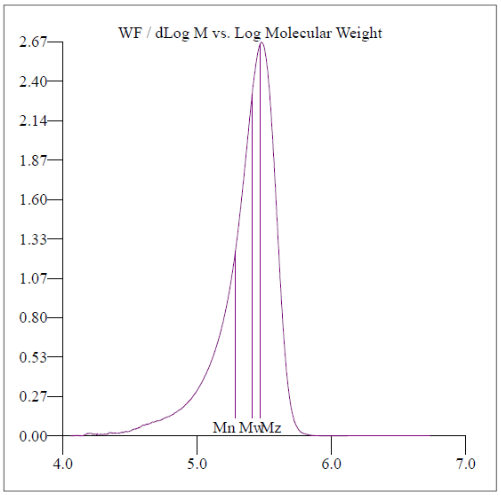

Figure S24. GPC of polyethylene. (Table 1, Entry 14)

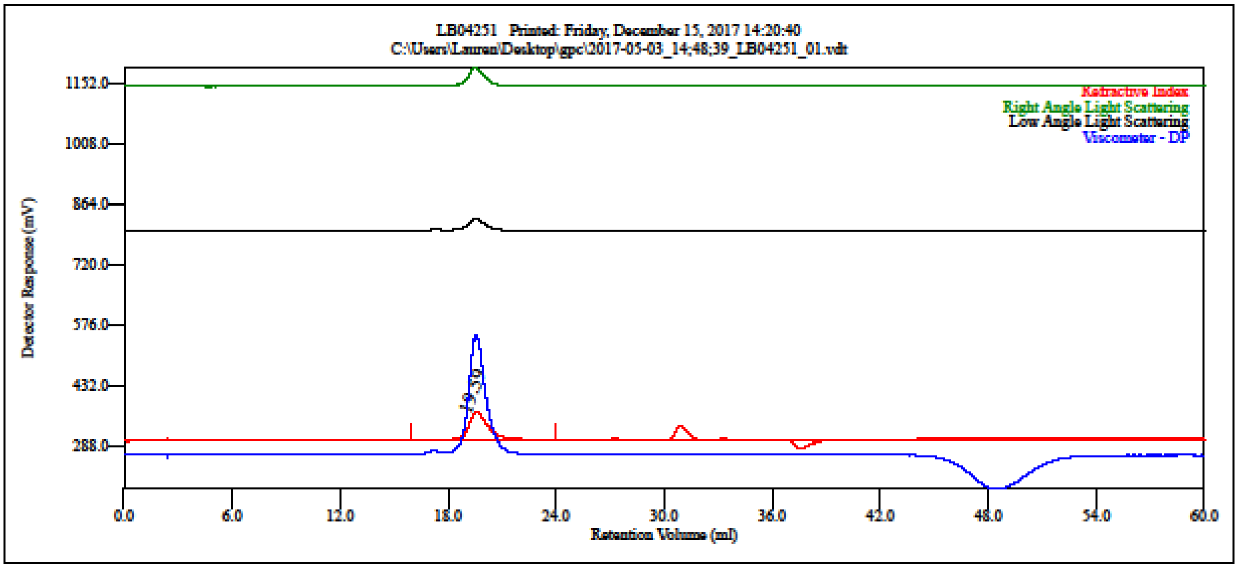

Multi-Detectors - Homopolymers : Results

Peak RV - (ml)	19.587
Mn - (Daltons)	42,913
Mw - (Daltons)	52,280
Mz - (Daltons)	62,992
Mp - (Daltons)	54,955
Mw / Mn	1.218
Percent Above Mw: 0	100.000
Percent Below Mw: 0	0.000
IV - (dl/g)	1.1994
Rh(w) - (nm)	9.848
Rg(w) - (nm)	No Calc
Wt Fr (Peak)	1.000
Mark-Houwink a	0.639
Mark-Houwink logK	-2.930
Branches	0.000
Branch Freq.	0.000
RI Area - (mvml)	80.05
UV Area - (mvml)	0.00
RALS Area - (mvml)	53.32
LALS Area - (mvml)	34.70
IVDP Area - (mvml)	328.75

Sample Parameters	Input	Calculated
Sample Conc - (mg/ml)	2.120	0.000
Sample Recovery (%)	0.000	76.218
dn/dc - (ml/g)	0.1040	0.0000
dA/dc - (ml/g)	1.0000	0.0000

Annotation	
Method File	LONG GROUP 4-28-17-0000.vcm
Limits File	
Date Acquired	May 03, 2017 - 14:48:39
Solvent	TCB
Acquisition Operator	admin : Administrator
Calculation Operator	admin : Administrator
Column Set	CLM6210 - HT X 3
System	System 1
Flow Rate - (ml/min)	1.000
Inj Volume - (ul)	200.0
Volume Increment - (ml)	0.00333
Detector Temp. - (deg C)	160.0
Column Temp. - (deg C)	160.0
OmniSEC Build Number	406

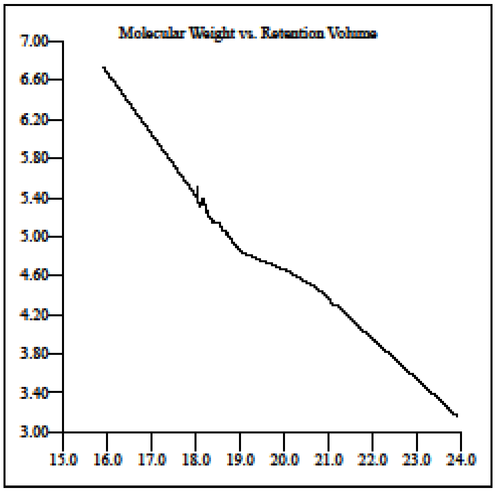

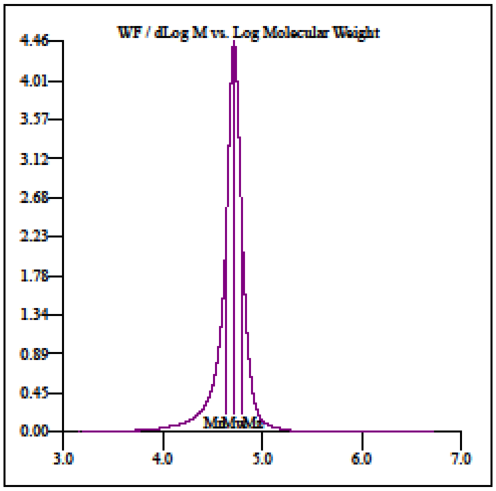

Figure S25. GPC of polyethylene. (Table 2, Entry 1)

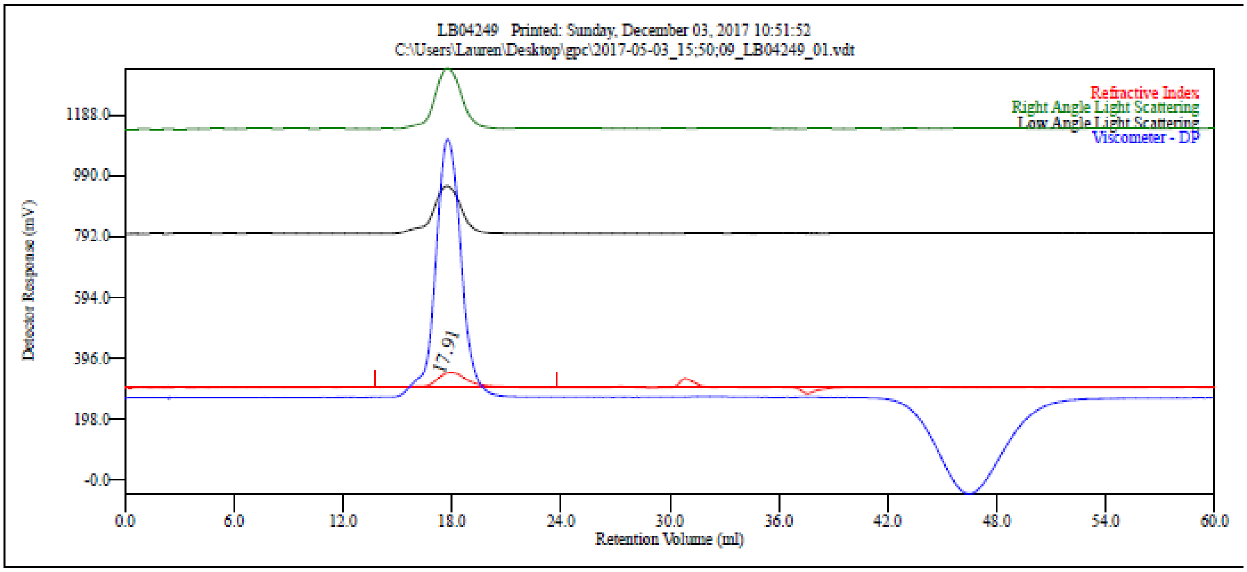

Multi-Detectors - Homopolymers : Results

Peak RV - (ml)	17.910
Mn - (Daltons)	279,976
Mw - (Daltons)	353,742
Mz - (Daltons)	573,672
Mp - (Daltons)	353,276
Mw / Mn	1.263
Percent Above Mw: 0	100.000
Percent Below Mw: 0	0.000
IV - (dl/g)	4.2641
Rh(w) - (nm)	28.002
Rg(w) - (nm)	37.298
Wt Fr (Peak)	1.000
Mark-Houwink a	0.784
Mark-Houwink logK	-3.714
Branches	0.000
Branch Freq.	0.000
RI Area - (mvml)	87.64
UV Area - (mvml)	0.00
RALS Area - (mvml)	335.01
LALS Area - (mvml)	274.93
IVDP Area - (mvml)	1384.71

Sample Parameters	Input	Calculated
Sample Conc - (mg/ml)	2.520	0.000
Sample Recovery (%)	0.000	68.350
dn/dc - (ml/g)	0.1040	0.0000
dA/dc - (ml/g)	1.0000	0.0000

Annotation	
Method File	Long Group 1-9-16-0000.vcm
Limits File	
Date Acquired	May 03, 2017 - 15:50:09
Solvent	TCB
Acquisition Operator	admin : Administrator
Calculation Operator	admin : Administrator
Column Set	CLM6210 - HT X 3
System	System 1
Flow Rate - (ml/min)	1.000
Inj Volume - (ul)	200.0
Volume Increment - (ml)	0.00333
Detector Temp. - (deg C)	160.0
Column Temp. - (deg C)	160.0
OmniSEC Build Number	406

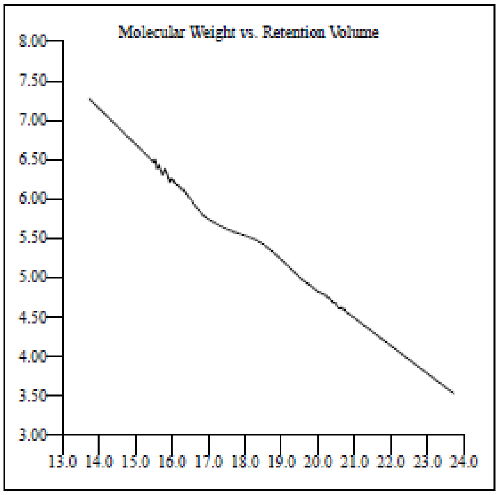

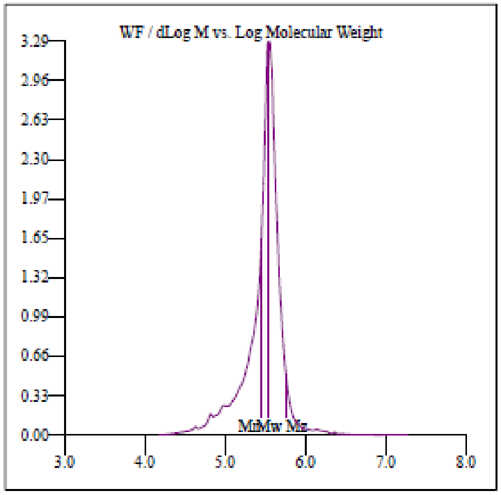

Figure S26. GPC of polyethylene. (Table 2, Entry 3)

LB04250 Printed: Sunday, December 03, 2017 10:53:31
C:\Users\Lauren\Desktop\gpc\2017-05-03_13;47;10_LB04250_01.vdt

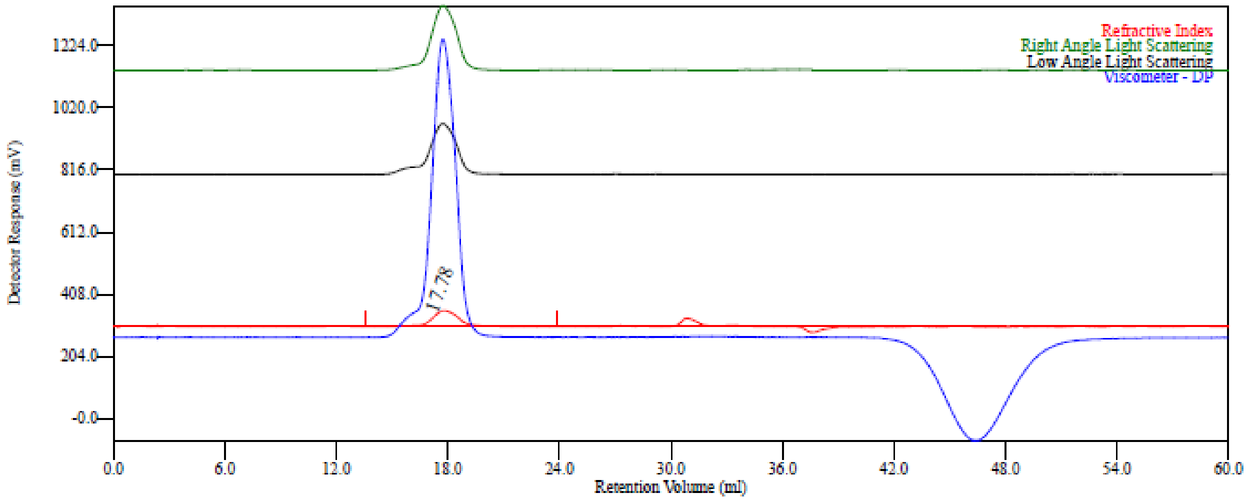

Molecular weight data for peak 1 is non-monotonic. Please read Help - FAQ entry.

Multi-Detectors - Homopolymers : Results

Peak RV - (ml)	17.777
Mn - (Daltons)	352,561
Mw - (Daltons)	432,344
Mz - (Daltons)	529,745
Mp - (Daltons)	405,716
Mw / Mn	1.226
Percent Above Mw: 0	100.000
Percent Below Mw: 0	0.000
IV - (dl/g)	5.3770
Rh(w) - (nm)	32.741
Rg(w) - (nm)	42.823
Wt Fr (Peak)	1.000
Mark-Houwink a	0.720
Mark-Houwink logK	-3.336
Branches	0.000
Branch Freq.	0.000
RI Area - (mvml)	81.72
UV Area - (mvml)	0.00
RALS Area - (mvml)	330.21
LALS Area - (mvml)	282.65
IVDP Area - (mvml)	1485.35

Sample Parameters	Input	Calculated
Sample Conc - (mg/ml)	2.280	0.000
Sample Recovery (%)	0.000	78.066
dn/dc - (ml/g)	0.1040	0.0000
dA/dc - (ml/g)	1.0000	0.0000

Annotation	
Method File	LONG GROUP 3-19-17-0000.vcm
Limits File	
Date Acquired	May 03, 2017 - 13:47:10
Solvent	TCB
Acquisition Operator	admin : Administrator
Calculation Operator	admin : Administrator
Column Set	CLM6210 - HT X 3
System	System 1
Flow Rate - (ml/min)	1.000
Inj Volume - (ul)	200.0
Volume Increment - (ml)	0.00333
Detector Temp. - (deg C)	160.0
Column Temp. - (deg C)	160.0
OmniSEC Build Number	406

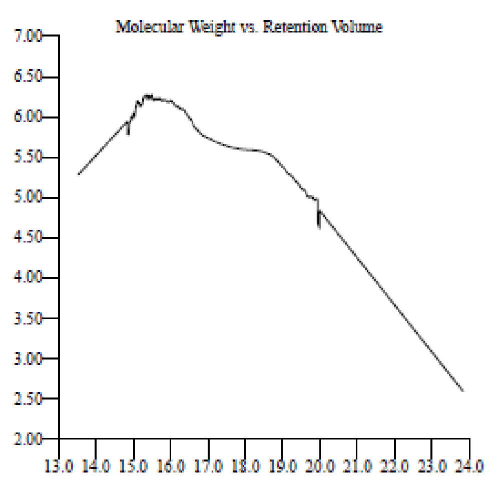

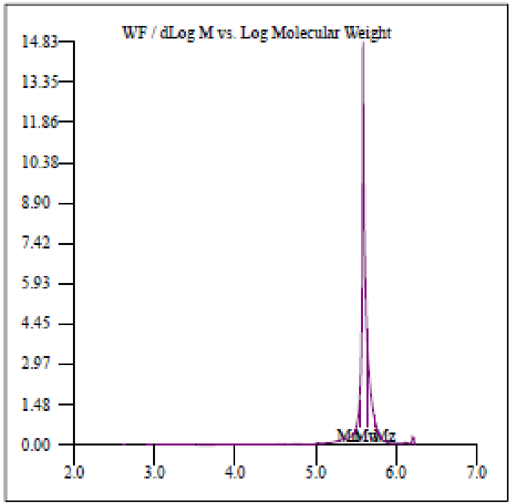

Figure S27. GPC of polyethylene. (Table 2, Entry 4)

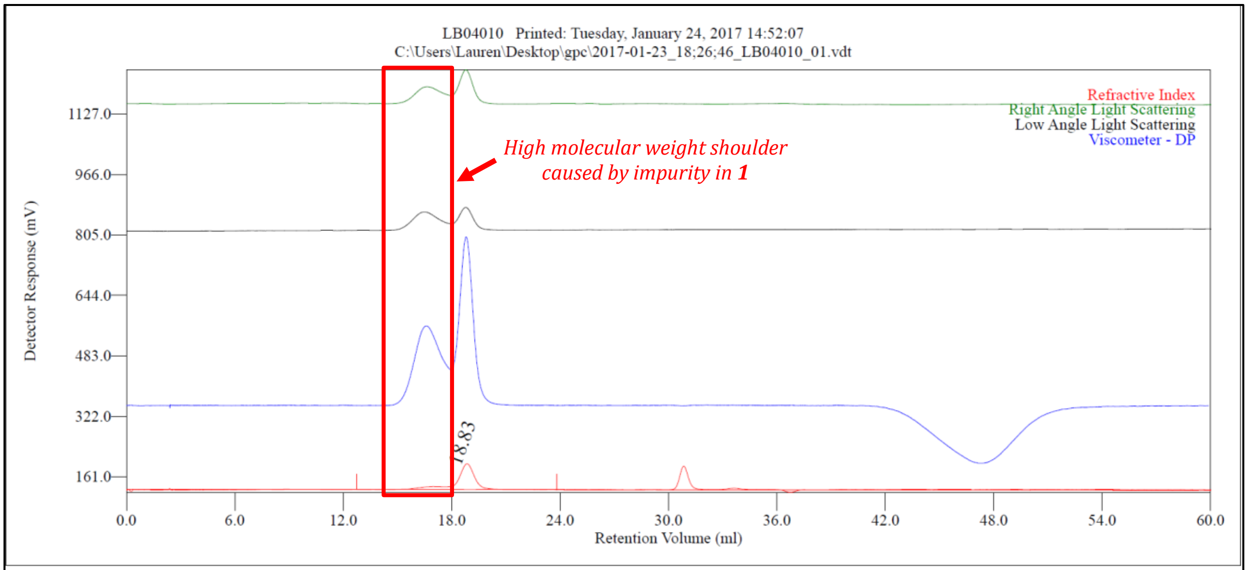

Molecular weight data for peak 1 is non-monotonic. Please read Help - FAQ entry.

Multi-Detectors - Homopolymers : Results

Peak RV - (ml)	18.833
Mn - (Daltons)	125,962
Mw - (Daltons)	233,363
Mz - (Daltons)	644,266
Mp - (Daltons)	109,749
Mw / Mn	1.853
Percent Above Mw: 0	100.000
Percent Below Mw: 0	0.000
IV - (dl/g)	3.1140
Rh(w) - (nm)	20.693
Rg(w) - (nm)	30.596
Wt Fr (Peak)	1.000
Mark-Houwink a	0.753
Mark-Houwink logK	-3.514
Branches	0.000
Branch Freq.	0.000
RI Area - (mvml)	77.16
UV Area - (mvml)	0.00
RALS Area - (mvml)	165.03
LALS Area - (mvml)	145.28
IVDP Area - (mvml)	789.68

Sample Parameters	Input	Calculated
Sample Conc - (mg/ml)	2.000	0.000
Sample Recovery (%)	0.000	75.821
dn/dc - (ml/g)	0.1040	0.0000
dA/dc - (ml/g)	1.0000	0.0000

Annotation	
Method File	Long Group 1-9-16-0000.vcm
Limits File	
Date Acquired	Jan 23, 2017 - 18:26:46
Solvent	TCB
Acquisition Operator	admin : Administrator
Calculation Operator	admin : Administrator
Column Set	CLM6210 - HT X 3
System	System 1
Flow Rate - (ml/min)	1.000
Inj Volume - (ul)	200.0
Volume Increment - (ml)	0.00333
Detector Temp. - (deg C)	160.0
Column Temp. - (deg C)	160.0
OmniSEC Build Number	406

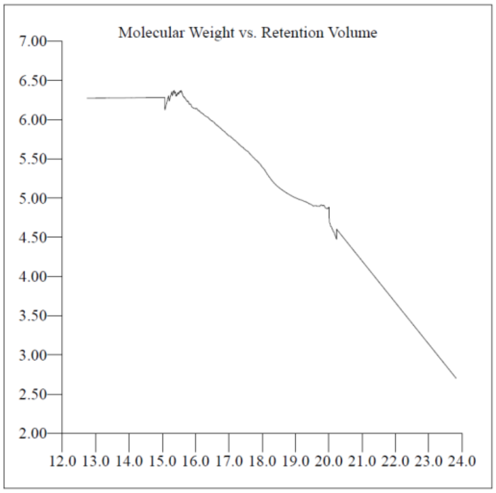

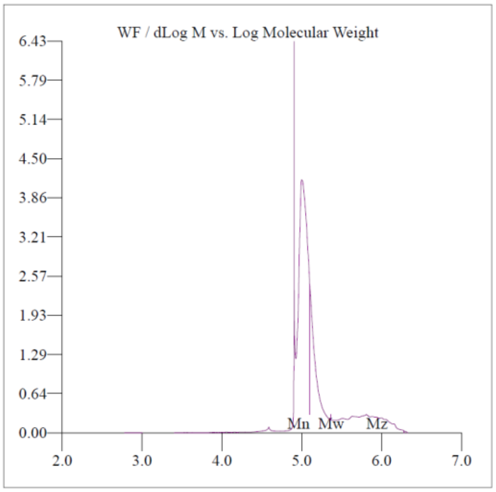

Figure S28. Representative GPC of polyethylene produced using complex **1** prior to rigorous ligand purification (*Note the presence of a high molecular weight shoulder*).

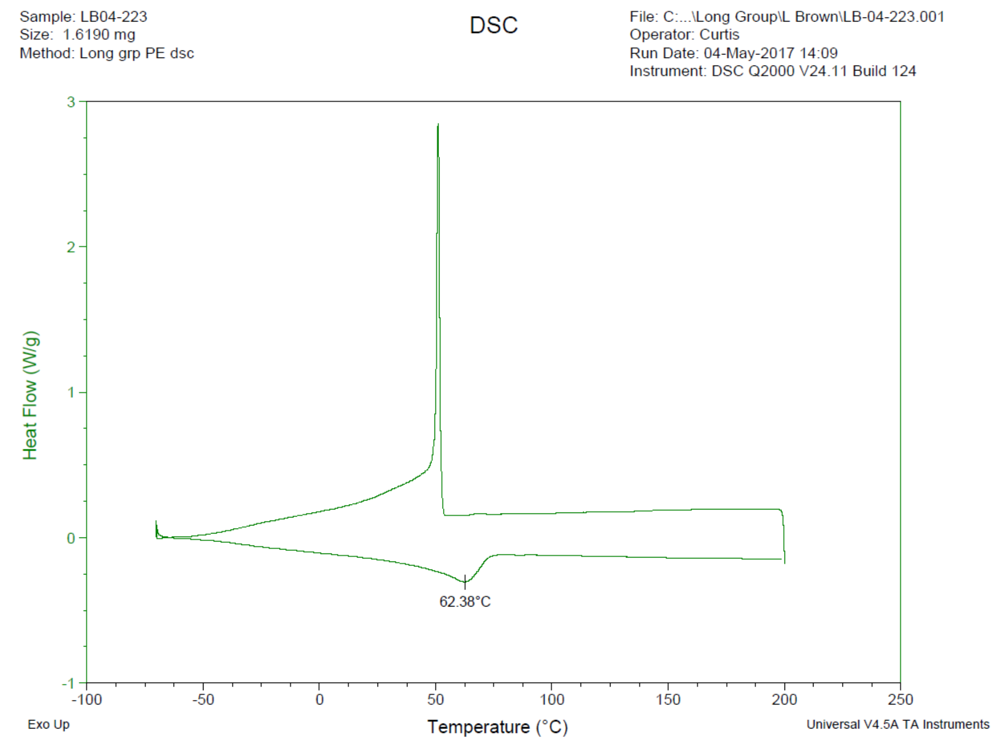

Figure S29. DSC of polyethylene. (Table 2, Entry 1)

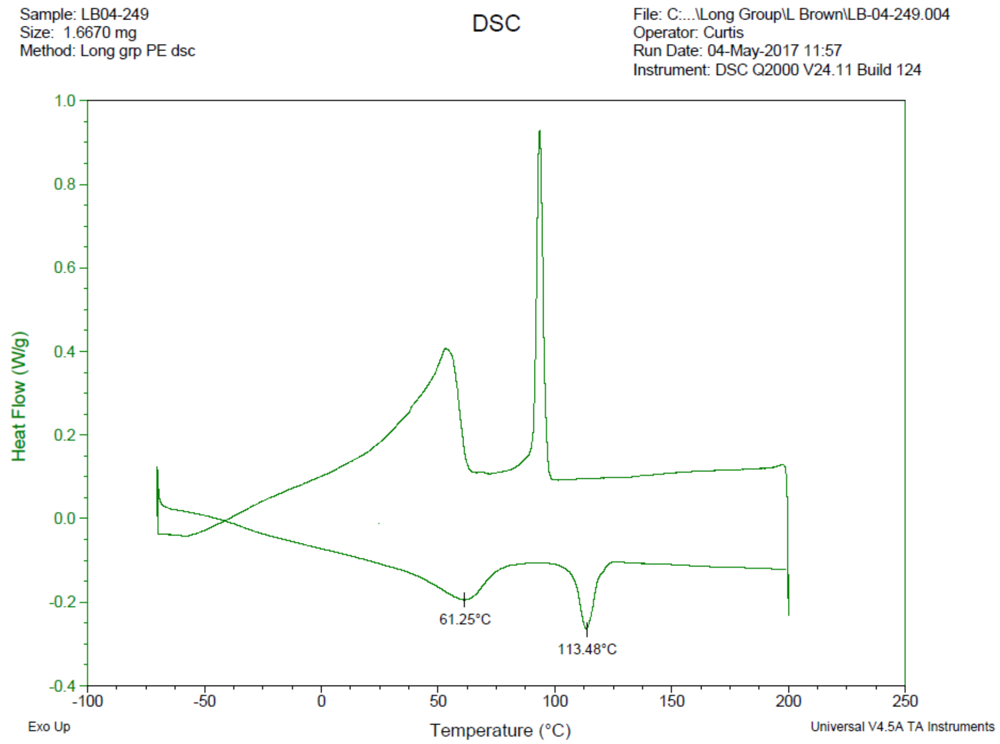

Figure S30. DSC of polyethylene. (Table 2, Entry 2)

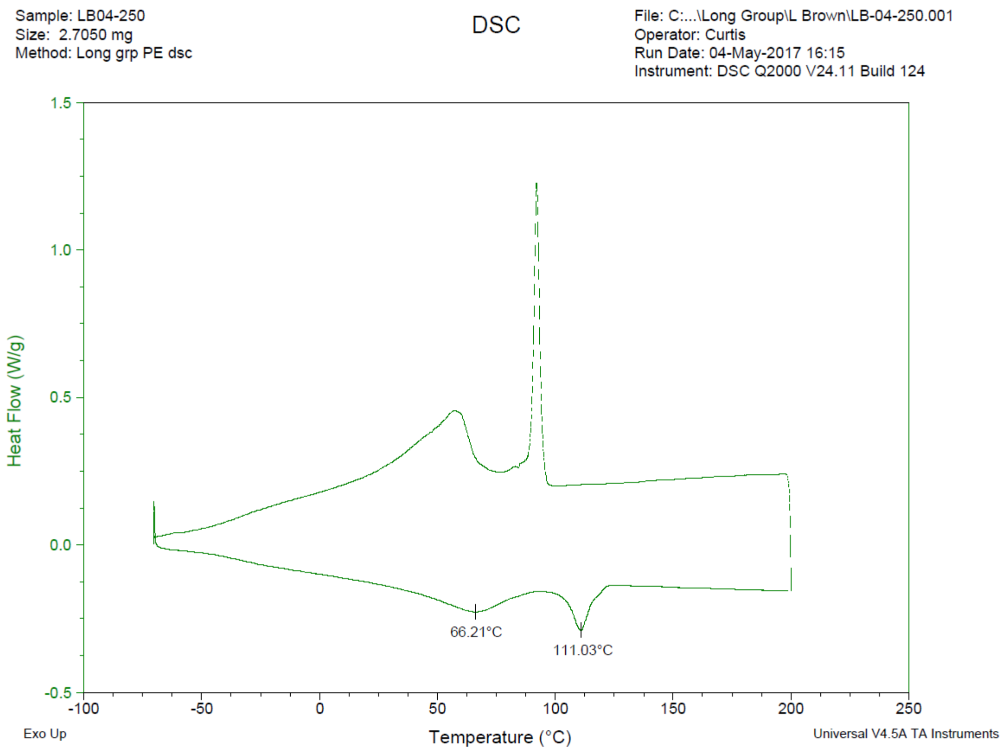

Figure S31. DSC of polyethylene. (Table 2, Entry 3)

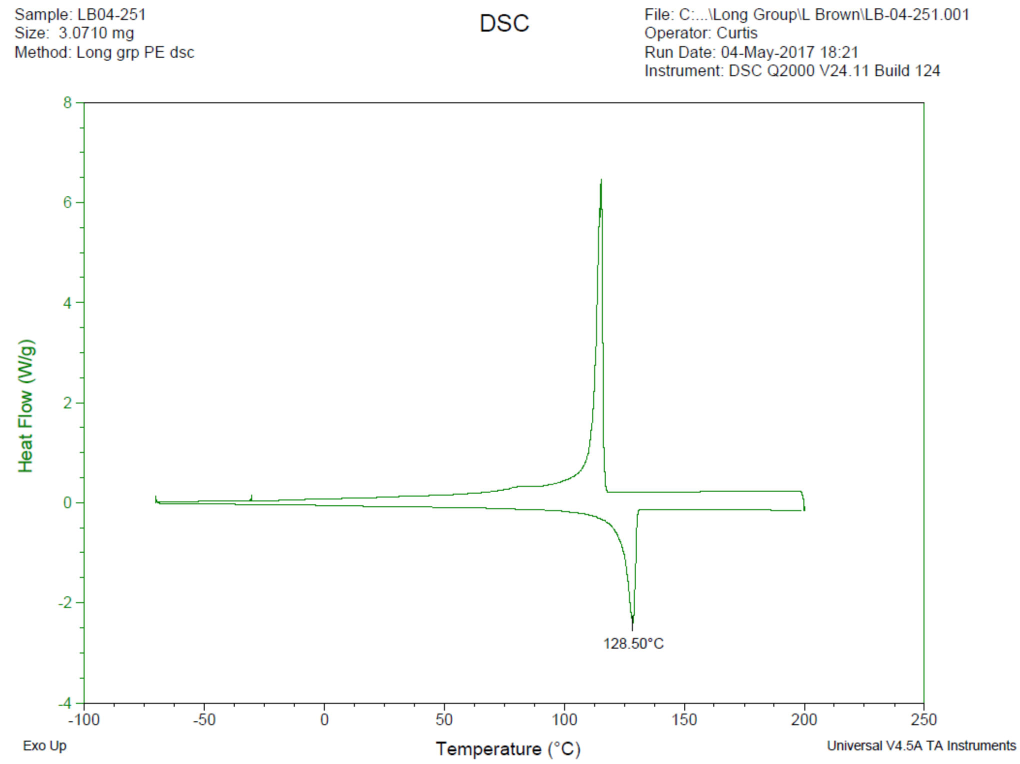

Figure S32. DSC of polyethylene. (Table 2, Entry 4)

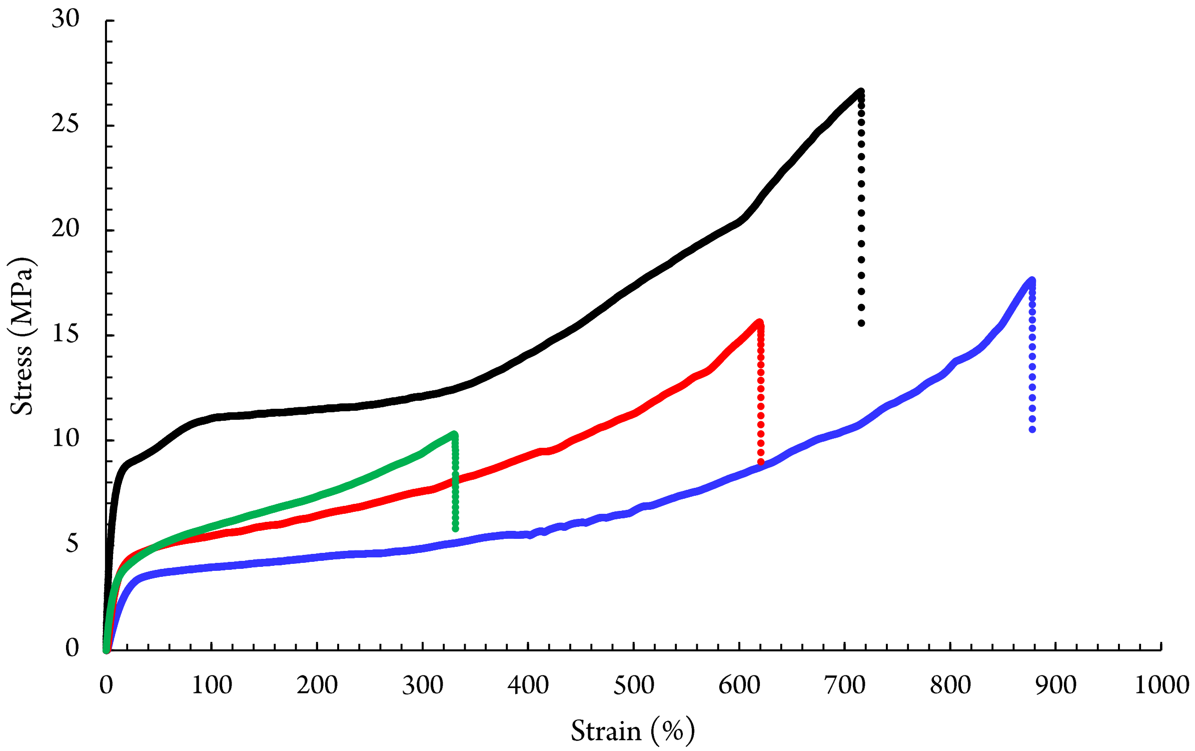

Figure S33. Plot of Stress versus Strain for polyethylene homopolymers and block copolymers. Key: ● = Table 2, entry 1; ● = Table 2, entry 2; ● = Table 2, entry 3; ● = Table 2, entry 4.
